# Trajectory inference and parameter estimation in stochastic models with temporally aggregated data

**DOI:** 10.1007/s11222-017-9779-x

**Published:** 2017-10-24

**Authors:** Maria Myrto Folia, Magnus Rattray

**Affiliations:** Division of Informatics, Imaging and Data Sciences, Faculty of Biology, Medicine and Health, University of Manchester, Manchester, UK

**Keywords:** Linear noise approximation, Stochastic systems biology, Time aggregation, Kalman filter

## Abstract

Stochastic models are of fundamental importance in many scientific and engineering applications. For example, stochastic models provide valuable insights into the causes and consequences of intra-cellular fluctuations and inter-cellular heterogeneity in molecular biology. The chemical master equation can be used to model intra-cellular stochasticity in living cells, but analytical solutions are rare and numerical simulations are computationally expensive. Inference of system trajectories and estimation of model parameters from observed data are important tasks and are even more challenging. Here, we consider the case where the observed data are aggregated over time. Aggregation of data over time is required in studies of single cell gene expression using a luciferase reporter, where the emitted light can be very faint and is therefore collected for several minutes for each observation. We show how an existing approach to inference based on the linear noise approximation (LNA) can be generalised to the case of temporally aggregated data. We provide a Kalman filter (KF) algorithm which can be combined with the LNA to carry out inference of system variable trajectories and estimation of model parameters. We apply and evaluate our method on both synthetic and real data scenarios and show that it is able to accurately infer the posterior distribution of model parameters in these examples. We demonstrate how applying standard KF inference to aggregated data without accounting for aggregation will tend to underestimate the process noise and can lead to biased parameter estimates.

## Introduction

Stochastic differential equations (SDEs) are used to model the dynamics of processes that evolve randomly over time. SDEs have found a range of applications in finance (e.g. stock markets, Hull 2009), physics (e.g. statistical physics, Gardiner 2004) and biology (e.g. biochemical processes, Wilkinson 2011). Usually, the coefficients (model parameters) of SDEs are unknown and have to be inferred using observations from the systems of interest. Observations are typically partial (e.g. collected at discrete times for a subset of variables), corrupted by measurement noise, and may also be aggregated over time and/or space. Given these observed data, our task is to infer the process trajectory and estimate the model parameters.

A motivating example of stochastic aggregated data comes from biology and more specifically from luminescence bioimaging, where a luciferase reporter gene is used for studying gene expression inside a cell (Spiller et al. 2010). The luminescence intensity emitted from the luciferase experiments is collected from single cells and is integrated over a time period (in certain cases up to 30 min, Harper et al. 2011) and then recorded as a single data point. In this paper, we consider the problem of inferring SDE model parameters given temporally aggregated data of this kind.

Imaging data from single cells are highly stochastic due to the low number of reactant molecules and the inherent stochasticity of cellular processes such as gene transcription or protein translation. The chemical master equation (CME) is widely used to describe the evolution of biochemical reactions inside cells stochastically (Gillespie 1992). Exact inference with the CME is rare and, even when possible, computationally prohibitive. In Golightly and Wilkinson (2005), the authors perform inference using a diffusion approximation of the CME, resulting in a nonlinear SDE. The linear noise approximation (LNA) (Kampen 2007) has been used as an alternative approximation of the CME which is valid for a sufficiently large system (Komorowski et al. 2009; Fearnhead et al. 2014). According to the LNA, the system is decomposed into a deterministic and a stochastic part. The latter is described by a linear SDE of the following form:

1 $$\begin{aligned} \mathrm{d}X_t = a_tX_t \mathrm{d}t + b_t \mathrm{d}W_t, \end{aligned}$$

where $X_t$ is a *d*-dimensional process, $a_t$ is a $(d\times d)$-matrix-valued function, $W_t$ is an *m*-dimensional Wiener process, and $b_t$ a $(d\times m)$ matrix-valued function.

Given an initial condition $X_0 = c$, Eq. (1) has the following known solution (Arnold 1974):

2 $$\begin{aligned} X_t = \varPhi _tc+\varPhi _t\int _{t_0}^t\varPhi _s^{-1}b_s\mathrm{d}W_s \ , \end{aligned}$$

where $\varPhi _t$ is the fundamental matrix of the homogeneous equation $\mathrm{d}X_t = a_tX_t\mathrm{d}t$. Note that the right integral in Eq. (2) is a Gaussian process, as it is an integral of a non-random function with respect to $W_t$ (Arnold 1974). If we further assume that the initial condition *c* is normally distributed or constant, Eq. (2) gives rise to a Gaussian process. Additionally, the solution of a (linear) SDE is a Markov process (Arnold 1974). These properties of linear SDEs (of the form of Eq. (1)) are highly desirable when carrying out inference.

The approaches above do not treat the aggregated nature of luciferase data in a principled way but instead assume that the data are proportional to the quantity of interest at the measurement time (Harper et al. 2011; Komorowski et al. 2009). Here, we build on the work of Komorowski et al. (2009) and Fearnhead et al. (2014) and extend it to the case of aggregated data. Since we are using the LNA, the problem is equivalent to a parameter inference problem for the time integral of a linear SDE as in Eq. (1): $\int _{t_0}^t X(u)\mathrm{d}u$. We follow a Bayesian approach, where the likelihood of our model is computed using a continuous-discrete Kalman filter (Särkkä 2006) and parameter inference is achieved using an MCMC algorithm. The paper is structured as follows: we first provide a description of the LNA as an approximation of the CME and introduce the integral of the LNA for treating temporally aggregated observations. We then describe a Kalman filter framework for performing inference with the LNA and its integral. Finally, we apply our method in three different examples. The Ornstein–Uhlenbeck process has been picked as a system where we can study its exact solutions. The Lotka–Volterra model was selected as an example of a nonlinear system with partial observations. The translation inhibition model was used to demonstrate our method with real data.

## The linear noise approximation and its integral

The CME can be used to model biochemical reactions inside a cell. It is essentially a forward Kolmogorov equation for a Markov process that describes the evolution of a spatially homogeneous biochemical system over time.

Assume a biochemical reaction network consisting of *N* chemical species ${\mathcal {X}_1}, \ldots ,{\mathcal {X}_N}$ in a volume ${\varOmega }$ and *v* reactions ${R_1}, \ldots ,{R_v}$. The usual notation for such a network is given below:

$${R_1}$$ : $${p_{11}\mathcal {X}_1}$$ + $${p_{12}\mathcal {X}_2} + \cdots + {p_{1N}\mathcal {X}_N}$$ $${\rightarrow }$$ $${q_{11}\mathcal {X}_1}$$ + $${q_{12}\mathcal {X}_2} +\cdots + {q_{1N}\mathcal {X}_N}$$ $${R_2}$$ : $${p_{21}\mathcal {X}_1}$$ + $${p_{22}\mathcal {X}_2} + \cdots + {p_{2N}\mathcal {X}_N}$$ $${\rightarrow }$$ $${q_{21}\mathcal {X}_1}$$ + $${q_{22}\mathcal {X}_2} + \cdots + {q_{2N}\mathcal {X}_N}$$ ... $${R_v}$$ : $${p_{v1}\mathcal {X}_1}$$ + $${p_{v2}\mathcal {X}_2} +\cdots + {p_{vN}\mathcal {X}_N}$$ $${\rightarrow }$$ $${q_{v1}\mathcal {X}_1}$$ + $${q_{v2}\mathcal {X}_2} + \cdots + {q_{vN}\mathcal {X}_N}$$

where ${\varvec{X}}$ = ${(\mathcal {X}_1,\ldots ,\mathcal {X}_N)^T}$ represents the number of chemical species (we assume molecules) and ${\varvec{x}}$ = ${\frac{\varvec{X}}{\varOmega }}$ is the concentration of molecules. We denote with *P* the ${v\times u}$ matrix whose elements are given by ${p_{ij}}$ and *Q* the ${v\times u}$ matrix with elements ${q_{ij}}$. We define the *stoichiometry matrix* *S* as ${S = (Q - P)^T}$. The probability of a reaction taking place in ${[t, t+\mathrm{d}t)}$ is given by the vector of reaction rates ${h_j(x,\varOmega , t)\varOmega \mathrm{d}t}$.

The probability ${p(\varvec{X},t)}$ that the system is in state ${\varvec{X}}$ at time *t* is given by the CME:

3 $$\begin{aligned} \begin{aligned} \frac{\mathrm{d}p(\varvec{X},t)}{\mathrm{d}t}&= \varOmega \sum \limits _{i=1}^v [h_i(\varvec{X}- S^{(i)},\varOmega , t)p(\varvec{X}-S^{(i)},t) \\&\quad - h_i(\varvec{X},\varOmega , t)p(\varvec{X},t)] \ . \end{aligned} \end{aligned}$$

However, as mentioned before, exact inference with the CME, even when possible, is computationally prohibitive. We use the LNA as an approximation of the CME due to its successful application in Komorowski et al. (2009) and Fearnhead et al. (2014). The state of the system ${\varvec{X}}$ is expected to have a peak around the macroscopic value of order $\varOmega$ and fluctuations of order $\varOmega ^{1/2}$ such that $X_t = \varOmega \phi _t + \varOmega ^{1/2}\xi _t$. This way the system is decomposed to the deterministic part $\phi _t$ and the stochastic part $\xi _t$. The LNA arises as a Taylor expansion of the CME in powers of the volume $\varOmega$; for a detailed derivation the reader is referred to Kampen (2007) and Elf and Ehrenberg (2003). By collecting terms of order $\varOmega ^{1/2}$, we obtain the deterministic part of the system, namely the macroscopic rate equations $\phi _i$, where *i* stands for the *i*th species:

4 $$\begin{aligned} \frac{\mathrm{d}\phi _i}{\mathrm{d}t} = S_ih(\phi _t,\varOmega , t) \ . \end{aligned}$$

Terms of order $\varOmega ^0$ give us the stochastic part of the system:

5 $$\begin{aligned} d\xi _t = A_t\xi _t\mathrm{d}t + E_t\mathrm{d}W \ , \end{aligned}$$

where, $A_t = SF_t$ and $F_{ij} = \frac{\partial h_j(\phi _t,\varOmega , t)}{\partial \phi _i(t) }$, while ${EE_t}^{T} = Sdiag(h(\phi _t,\varOmega , t))S^T$. Equation (5) is a linear SDE of the form of Eq. (1). Its solution is a Gaussian Markov process, provided that we have an initial condition that is a constant or a Gaussian random variable. The ordinary differential equations (ODEs) that describe the mean and variance of this Gaussian process are given by Arnold (1974):

6 $$\begin{aligned} \frac{\mathrm{d}m_t}{\mathrm{d}t}= & {} A_tm_t \ , \end{aligned}$$

7 $$\begin{aligned} \frac{\mathrm{d}V_t}{\mathrm{d}t}= & {} V_t{A_t}^T+A_tV_t+{EE_t}^T \ . \end{aligned}$$

Note that if we set the initial condition of $m_0=0$, then Eq. (6) will lead to $m_t = 0$ at all times. We will make the assumption that, at each observation point, $m_t$ is reset to zero since it can be beneficial for inference as discussed in Fearnhead et al. (2014) and Giagos (2010).

In what follows we will assume, without loss of generality, that the volume ${\varOmega }=1$, i.e. the number of molecules equals the concentration of molecules and thus,

8 $$\begin{aligned} X_t= \phi _t + \xi _t \ . \end{aligned}$$

Equation (8) is the sum of a deterministic and a Gaussian term; consequently, it will also be normally distributed. By taking its expectation and variance, we have that $X_t|X_0 \sim N(\phi _t + m_t,V_t)$ which, according to the initial condition $m_0=0$, leads to $X_t|X_0 \sim N(\phi _t,V_t)$.

We are now interested in the integral of Eq. (8), as this will allow us to model the aggregated data,

9 $$\begin{aligned} H_t = \int _{t_0}^t X_u\mathrm{d}u = \int _{t_0}^t \phi _u\mathrm{d}u + \int _{t_0}^t \xi _u\mathrm{d}u = I_t + Q_t \ . \end{aligned}$$

The deterministic part of this aggregated process is given by *I*(*t*), and the stochastic part is given by *Q*(*t*). Subsequently, we have the following ODEs:

10 $$\begin{aligned} \frac{\mathrm{d}I_t}{\mathrm{d}t}= & {} \frac{d}{\mathrm{d}t} \int _{t_0}^t \phi _t\mathrm{d}u = \phi _t \ , \end{aligned}$$

11 $$\begin{aligned} \frac{dQ_t}{\mathrm{d}t}= & {} \xi _t \ . \end{aligned}$$

Here, $Q_t$ will also follow a Gaussian process (as it is the integral of a Gaussian process) so we need to compute its mean and variance. The ODEs for the mean, variance and $\mathbb {E}[Q_t\xi _t^T]$ are given below; their proofs can be found in “Appendix A.1”:

12 $$\begin{aligned} \frac{\mathrm{d}{E}[Q_{t}]}{\mathrm{d}t}= & {} \mathbb {E}[\xi _t] = 0, \end{aligned}$$

13 $$\begin{aligned} \frac{d\mathrm {Var}[Q_t]}{\mathrm{d}t}= & {} \mathbb {E}[Q_t\xi _t^T] + \mathbb {E}[\xi _tQ_t^T] \ ,\end{aligned}$$

14 $$\begin{aligned} \frac{d\mathbb {E}[Q_t\xi _t^T]}{dt}= & {} \mathbb {E}[Q_t\xi _t^T]A(t)^T + V_t \ . \end{aligned}$$

Note that $Q_t$ is not Markovian since knowledge of its history is not sufficient to determine its current state. However, jointly with $\xi _t$ it forms a bivariate Gaussian Markov process, that is characterised by the following linear SDE:

15 $$\begin{aligned} d\begin{bmatrix} \xi _t\\Q_t\end{bmatrix}= & {} \begin{bmatrix} A_t&0\\1&0\end{bmatrix}\begin{bmatrix} \xi _t\\Q_t\end{bmatrix}dt+\begin{bmatrix} E_t\\0 \end{bmatrix}dW_t \ , \nonumber \\ \begin{bmatrix} \xi _0\\Q_0\end{bmatrix}= & {} \begin{bmatrix} 0\\0\end{bmatrix} \ . \end{aligned}$$

From Eq. (15) we have that $\xi _t, Q_t$ are jointly Gaussian and, consequently, their marginals are also normally distributed. Thus, according to (9) $H_t|H_0,X_0 \sim N(\mu _t, \varSigma _t)$ with $\mu _t = I_t$ and $\varSigma _t = V[Q_t]$.

## Kalman filter for the LNA and its integral

The classical filtering problem is concerned with the problem of estimating the state of a linear system given noisy, indirect or partial observations (Kalman 1960). In our case, the state is continuous and is described by Eq. (8) while the observations are collected at discrete time points with or without Gaussian noise. For this reason, we refer to it as the continuous-discrete filtering problem (Jazwinski 1970; Särkkä 2006).

First, we consider the case where observations are taken from the process $X_t$ and not from its integral $H_t$. In that case, the observation process is given by $y_t = P_tX_t + \epsilon _t$ where $\epsilon _t \sim N(0,R)$ and accounts for technical noise. The observability matrix $P_t$ is used to deal with the partial observability of the system, for example, if we have two species $X_1$, $X_2$ and we observe only $X_1$, $P = [1,0]^\mathrm{T}$.

Following the Kalman filter (KF) methodology, we need to define the following quantities:

- Prior: $$p(X_0)$$.
- Predictive distribution: $p(X_t|y_{1:t-1})$, where $y_{1:t-1}$ refers to the observations at discrete points up to time $t-1$.
- Posterior or Update distribution: $p(X_t|y_{1:t})$.

The predictive distribution is given by $X_t|y_{1:t-1} \sim N(\mu _{1t}^-,V_{t}^-)$, where $\mu _{1t}^-$ and $V_{t}^-$ are found by integrating forward for $[t,t-1]$ Eqs. (4) and (7) initialised at the posterior mean $\mu _{1t-1}$ and variance $V_{t-1}$. In our case, the mean of the stochastic part is initialised at 0, so $\mu _{1t}$ corresponds to the deterministic part $\phi _t$. By updating the deterministic solution at each observation point, we achieve a better estimate, as the ODE solution can become a poor approximation over long periods of time. The posterior distribution $p(X_t|y_{1:t}) = N(\mu _{1t}, V_t)$ corresponds to the standard posterior distribution of a discrete KF and the updated $\mu _{1t}$ and $V_t$ are given in “Appendix A.3”. This case has been thoroughly studied in Fearnhead et al. (2014).

We consider now the case where the state $X_t$ is being observed through the integrated process $H_t$, such that the observation process is given by $y_t = P_tH_t + \epsilon _t$ and $\epsilon _t \sim N(0,R)$. Again, we need to define a prior distribution as well as calculate the predictive and posterior distributions for the system that we are studying.

The predictive distribution of our system is given by $$p\Big (\begin{bmatrix}X_t\\H_t\end{bmatrix}|y_{1:t-1}\Big ) = N\Big (\begin{bmatrix} \mu _{1t}^-\\\mu _{2t}^-\end{bmatrix}, \begin{bmatrix} V_{t}^-&{C_{t}^-}^T\\C_{t}^-&\varSigma _{t}^-\end{bmatrix} \Big )$$, where $C_t = \mathbb {E}[Q_tM_t^T]$. For this step, we need to integrate forward the ODEs (4), (10), (7), (13) and (14) with the appropriate initial conditions as seen in Algorithm 1. Note that the integrated process $H_t$ needs to be reset to 0 at each observation point in order to capture correctly the ‘area under graph’ of the underlying process $X_t$.

To compute the posterior distribution $p(X_t|y_{1:t})$, we look at the joint distribution of $(H_t,X_t,y_t)$ conditioned on $y_{1:t-1}$:

16 $$\begin{aligned} \begin{aligned}&\begin{bmatrix} X_t\\H_t\\y_t\end{bmatrix}|y_{1:t-1} \sim \\&\quad N\left( \begin{bmatrix} \mu _{1t}^-\\\mu _{2t}^-\\P_t\mu _{2t}^-\end{bmatrix},\begin{bmatrix} V_{t}^-&{C_{t}^-}^T&{C^-_t}^TP_t^T\\C_{t}^-&\varSigma _{t}^-&\varSigma _{t}^-P_t^T\\P_tC_{t}^-&P_t\varSigma _{t}^-&P_t \varSigma _{t}^-P_t^T+R_t\end{bmatrix}\right) \end{aligned} \end{aligned}$$

By using the lemma in “Appendix A.2” and using the corresponding blocks of the joint distribution (16), we can calculate the posterior mean and variance of $p(X_t|y_{1:t})$:

17 $$\begin{aligned} \mu _{1t}= & {} \mu _{1t}^- + {P_t{C_{t}^-}}^T(P_t\varSigma _{t}^ -P_t^T+R_t)^{-1}(y_t - P_t\mu _{2t}^-) \ , \nonumber \\ V_t= & {} V_{t}^- - {P_t{C_{t}^-}}^T(P_t\varSigma _{t}^-P_t^T+R_t)^{-1}P_t{C_{t}^-} \ . \end{aligned}$$

Since we are interested in parameter inference, we will need to compute the likelihood $L(\theta )$ of the system, where $\theta$ represents the parameter vector of the system:

18 $$\begin{aligned} L(\theta ) = p(y_1|\theta )\prod _{i=2}^tp(y_i|y_{1:i-1},\theta ) \ . \end{aligned}$$

The individual terms of the likelihood are given by $p(y_t|y_{1:t-1}) = N(P_t\mu _{2t}^-,P_t\varSigma _{t}^-P^T_t+R_t)$. Parameter inference is then straightforward either by using a numerical technique such as the Nelder–Mead algorithm to obtain the maximum likelihood (ML) parameters or using a Bayesian method such as a Metropolis-Hastings (MH) algorithm. The general procedure for performing inference using aggregated data is summarised in Algorithm 1. 
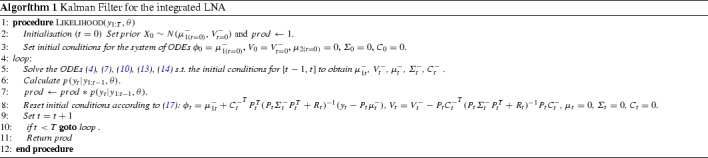


## The Ornstein–Uhlenbeck process

We first investigate the effect of integration in a one-dimensional, zero-mean OU process of the following form:

19 $$\begin{aligned} \mathrm{d}X_t = -\alpha X_t\mathrm{d}t + \sigma \mathrm{d}W_t, \end{aligned}$$

where $\alpha$ is the drift or decay rate of the process and $\sigma$ is the diffusion constant. Both of these parameters are assumed to be unknown, and we will try to infer them using the KF scheme that we have developed.

The OU process is a special case of a linear SDE (Eq. (1)), since its coefficients are time invariant, resulting in a stationary Gaussian–Markov process. Analytical solutions for both the OU and its integral exist (Gillespie 1996) and are presented in “Appendix A.4”. The results for the mean $m_t$ and variance $V_t$ of the OU, where $\Delta = t-t_0$, are given below:

20a $$\begin{aligned} m_t= & {} m_0e^{-\alpha \Delta } \ , \end{aligned}$$

20b $$\begin{aligned} V_t= & {} e^{-2\alpha \Delta }V_0+\frac{\sigma ^2}{2\alpha } \left( 1-e^{-2\alpha \Delta }\right) . \end{aligned}$$

The integral of Eq. (19) is given by $\mathrm{d}Y_t = X_t\mathrm{d}t$, and the mean, variance and covariance are given below,

21a $$\begin{aligned}&\mathbb {E}[y_t] = \frac{m_0}{\alpha }(1-e^{-\alpha \Delta }) \ , \end{aligned}$$

21b $$\begin{aligned}&\mathrm {Cov}(X_t,Y_t) = \frac{\sigma ^2}{2\alpha ^2} +\left( -\frac{\sigma ^2}{\alpha ^2}+\frac{V_0}{\alpha }\right) e^{-\alpha \Delta } \nonumber \\&\qquad \qquad \qquad \quad \,\,\, + \left( \frac{\sigma ^2}{2\alpha ^2}-\frac{V_0}{\alpha }\right) e^{-2\alpha \Delta } \ , \end{aligned}$$

21c $$\begin{aligned}&\mathrm {Var}[y_t] = \frac{\sigma ^2}{\alpha ^2} \Delta +\left( \frac{\sigma ^2}{2\alpha ^3}-\frac{V_0}{\alpha ^2} \right) \left( 1-e^{-2\alpha \Delta }\right) \nonumber \\&\qquad \qquad \quad \, +2\left( -\frac{\sigma ^2}{\alpha ^3}+\frac{V_0}{\alpha ^2} \right) (1-e^{-\alpha \Delta }) \ . \end{aligned}$$

We are interested in inferring the parameters $\alpha$ and $\sigma$ given observations from $Y_t$ at discrete times, where the interval $\Delta$ between two observations is constant. We will compare two approaches. First, we will assume that the data come directly from $X_t$ ignoring their aggregated nature and use the standard discrete–continuous KF, referred to as KF1. To make the comparison of this scenario fairer, we will normalise the observations by dividing with $\Delta$, which brings the observation close to an average value of the process, in an attempt to match the observations to data generated from the process $X_t$. In the second case, we will use the KF on the integrated process in analogy with Algorithm 1, which we will refer to as KF2. The case of inferring the parameters of an OU process using non-aggregated data with an MCMC algorithm has already been studied in Mbalawata et al. (2013).

$X_t$ will reach its stationary distribution after a time of order $\frac{1}{\alpha }$, which is given by $N(0,\frac{\sigma ^2}{2\alpha })$ (Gillespie 1992). However, the integrated process $Y_t$ is non-stationary since $\mathrm {Var}[y_t] \rightarrow \infty$ as $\Delta \rightarrow \infty$ . This already shows us that the two processes behave differently.

Since we are going to use the normalised observations from $Y_t$ with KF1, we will take a look at the normalised process $Z_t = \frac{1}{\Delta }Y_t$:

22a $$\begin{aligned}&\mathbb {E}[z_t] = \mathbb {E}[\frac{1}{\Delta }Y_t] = \frac{1}{\Delta }\mathbb {E}[y_t] = \frac{m_0}{\alpha \Delta } (1-e^{-\alpha \Delta }) \ , \end{aligned}$$

22b $$\begin{aligned}&\mathrm {Var}[z_t] = \mathrm {Var}[\frac{1}{\Delta }Y_t] = \frac{1}{\Delta ^2}\mathrm {Var}[y_t] = \nonumber \\&\qquad \qquad \quad \,\, \frac{\sigma ^2}{\alpha ^2\Delta }+\frac{1}{\Delta ^2} (\frac{\sigma ^2}{2\alpha ^3}-\frac{V_0}{\alpha ^2}) (1-e^{-2\alpha \Delta })+\nonumber \\&\qquad \qquad \quad \,\, +\frac{2}{\Delta ^2}(-\frac{\sigma ^2}{\alpha ^3} +\frac{V_0}{\alpha ^2})(1-e^{-\alpha \Delta }) \ . \end{aligned}$$

By taking the limit as $\Delta \rightarrow \infty$ in Eq. (22) and using L’Hospital’s rule we can show that $\mathbb {E}[z_t]\rightarrow 0$ and $\mathrm {Var}[z_t]\rightarrow 0$. So, the normalised process is again not approaching the stationary distribution of $X_t$.

We have generated aggregated data from the integral of an OU process with $\alpha = 4$ and $\sigma = 2$. To simulate data from $Y_t$, we need to first simulate data from $X_t$. This can be done in general by discretising the process and using the Euler–Maruyama algorithm. However, in the case of the OU process, we can also use an exact updating formula (see “Appendix A.6”). The aggregated data can then be collected using the discretised form $Y_{t+dt} = Y_t + X_tdt$ or a numerical integration method such as the trapezoidal rule over the indicated integration period. In “Appendix A.12” we have included plots of the OU process and the corresponding aggregated process.

We tested inference using KF1 with normalised data and KF2 with aggregated data. Results of parameter estimation using a standard random walk MH algorithm are presented in Table 1. Improper uniform priors over infinite range have been used on the log parameters, while different time intervals $\Delta$ have been considered. For each interval $\Delta$, we have sampled 100 observations from a single trajectory of an OU process with $\alpha =4$ and $\sigma = 2$ aggregated over the specified $\Delta$. For this example, we have assumed no observation noise. MCMC traceplots of $\alpha$ and $\sigma$ can be found in “Appendix A.13” (Figs. 6, 7) which indicate a good mixing of the chain and fast convergence. All chains were run for 50K iterations and 30K were discarded as burn-in. To verify the validity of the results, we have run nine more datasets, separately each time. An average over the ten datasets can be found in “Appendix A.7” (Table 5). As we can see, the estimates for KF1 deteriorate for larger $\Delta$. This is expected since the aggregated process diverges further from the OU process as $\Delta$ increases. Estimates remain good for KF2 even when $\Delta$ is large, although they become more uncertain, as can be witnessed by the increased standard deviations. Filtering results for KF1 and KF2 with aggregated data using the estimated parameter results for $\Delta = 1$ are given in “Appendix A.14”.

**Table 1:** Mean posterior ± 1 s.d. for $\alpha$ and $\sigma$ using a Metropolis-Hastings algorithm

$\Delta$	KF	$\alpha$	$\sigma$
0.1	KF1	$3.023\pm 0.235$	$1.891\pm 0.135$
0.5	KF1	$1.905\pm 0.141$	$1.256\pm 0.095$
1.0	KF1	$1.420\pm 0.102$	$0.868\pm 0.068$
2.0	KF1	$1.022\pm 0.075$	$0.540\pm 0.044$
0.1	KF2	$4.022\pm 0.295$	$2.113\pm 0.159$
0.5	KF2	$4.092\pm 0.335$	$2.311\pm 0.206$
1.0	KF2	$3.865\pm 0.368$	$2.234\pm 0.240$
2.0	KF2	$3.704\pm 0.513$	$2.082\pm 0.307$

Data were simulated from an OU process with $\alpha = 4$ and $\sigma = 2$

**Fig. 1:**
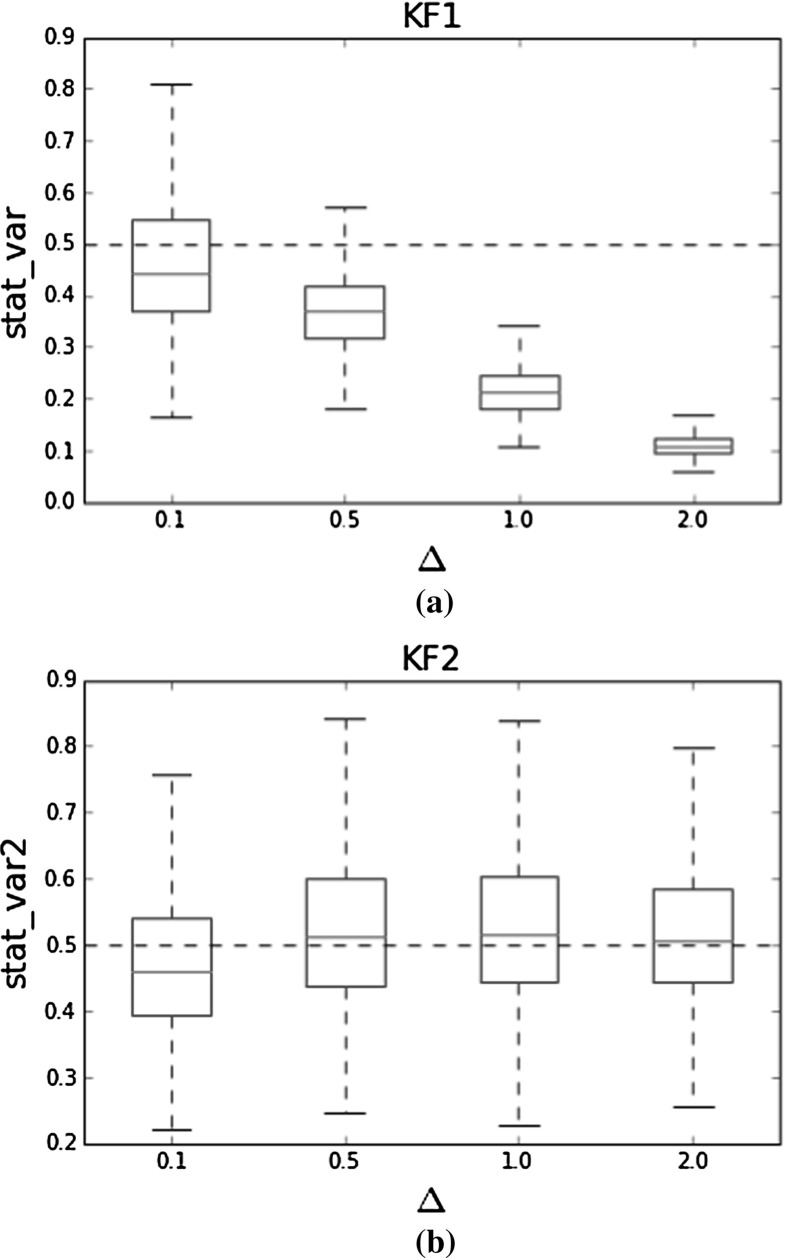
Boxplots of inferred stationary variance of the OU process for different $\Delta$. The simulated OU process has $\alpha = 4$ and $\sigma = 2$ corresponding to a stationary variance of 0.5, as indicated by the dotted horizontal line. The inferred stationary variance using KF1 tends to zero as $\Delta$ grows, but the stationary variance from KF2 is inferred correctly at all $\Delta$. **a** Boxplots of inferred stationary variance for different $\Delta$ using KF1. **b** Boxplots of inferred stationary variance for different $\Delta$ using KF2

It is of interest to investigate the inferred stationary variance of the OU process using KF1 and KF2. We have plotted the inferred stationary variances obtained by the MH for both KF1 and KF2 in Fig. 1. The boxplots are obtained using the average of 10 different datasets and correspond again to an OU process with $\alpha = 4$ and $\sigma = 2$, thus giving rise to a stationary variance of $\frac{\sigma ^2}{2\alpha } = 0.5$. When using the normalised aggregated data directly with KF1, we infer the wrong stationary variance of the underlying OU process which tends to zero as $\Delta$ becomes larger, consistent with the theoretical results from Eq. (22). Intuitively, we can attribute this behaviour to the fact that aggregated data have relatively smaller fluctuations, so that KF1 will tend to underestimate the process variance.

In this section, we have looked at an example of inferring the parameters of an SDE using aggregated data, and we have found that to obtain accurate results we need to explicitly model the aggregated process. As the observation intervals become larger, there is a greater mismatch between KF1 and KF2. In the next two sections, we will look at examples of more complex stochastic systems that must be approximated by the LNA and compare again inference results using KF1 and KF2.

## Lotka–Volterra model

We are now going to look at a system of two species that interact with each other according to three reactions

23a $$\begin{aligned}&X_1\xrightarrow {{\theta _1}}2X_1 \text { (prey production)} \end{aligned}$$

23b $$\begin{aligned}&X_1+X_2\xrightarrow {{\theta _2}}2X_2 \text { (predator production)}\end{aligned}$$

23c $$\begin{aligned}&X_2\xrightarrow {{\theta _3}}\oslash \text { (predator death)} \end{aligned}$$

The model represented by the biochemical reaction network (23) is known as the Lotka–Volterra model, with $X_1$ representing prey species and $X_2$ predator species. Although a simple model, it has been used as a reference model (Boys et al. 2008; Fearnhead et al. 2014) since it consists of two species, making it possible to observe it partially through one of the species and also provides a simple example of a nonlinear system.

The LNA can be used to approximate the dynamics and the resulting ODEs can be found in “Appendix A.8”. We want to compare parameter estimation results using KF1 and KF2. We collected aggregated data from a Lotka–Volterra model using the Gillespie algorithm. We assumed a known initial population of 10 prey species and 100 predator species. The parameters of the system for producing the synthetic data were set to $(\theta _1,\theta _2,\theta _3) = (0.5,0.0025,0.3)$, following (Boys et al. 2008). We have added Gaussian noise with standard deviation set to 3.0, and we assumed that the noise level was known for inference. Our goal was to infer the three parameters $(\theta _1,\theta _2,\theta _3)$ of the system using aggregated observations solely from the predator population.

**Fig. 2:**
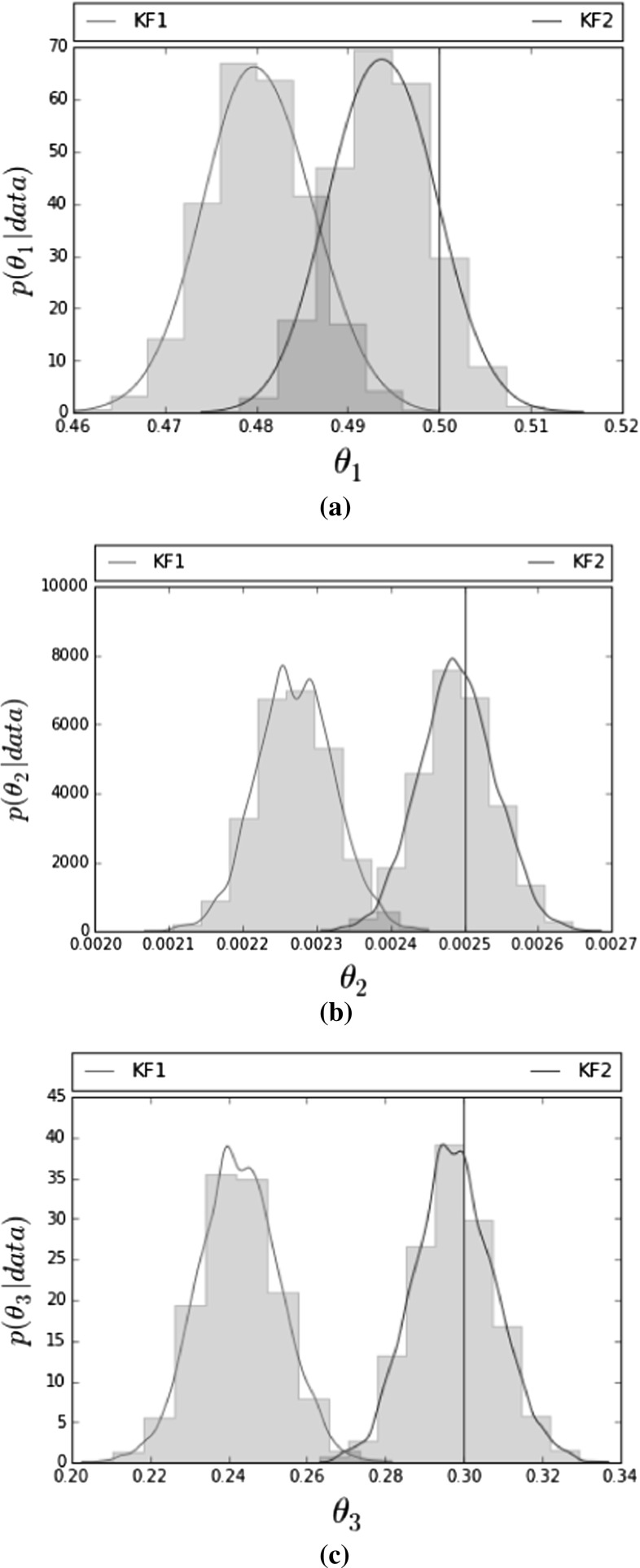
Posterior densities of $\theta _1,\theta _2,\theta _3$ from aggregated data using KF1 (red histogram) and KF2 (green histogram). **a** Posterior density of $\theta _1$. **b** Posterior density of $\theta _2$. **c** Posterior density of $\theta _3$

The Gillespie algorithm was run for 20 min. Data were aggregated and collected every 2 min resulting in 10 observations per sample. To infer the parameters, we assumed that we had 40 independent samples available. Since we assumed independence between the samples, we worked with the product of their likelihoods. In the ideal case of having complete data of a stochastic kinetic model the likelihood is conjugate to an independent gamma prior for the rate constants (Wilkinson 2011). The choice of Ga(2,10) with shape $= 2$ and rate $= 10$ gives a reasonable range for all three parameters and has also been used by Fearnhead et al. (2014). However, in this case the choice of prior is not important as the data dominate the posterior. We have run the same experiment using uninformative exponential priors Exp($10^{-4}$) that resulted in equivalent posterior distributions. Since we know that we want all parameters to be positive, we worked with a log transformation. MCMC convergence in this example is relatively slow and adaptive MCMC (Sherlock et al. 2010) was found to speed up convergence (see “Appendix A.9” for details). The adaptive MCMC was run for 30K iterations with 10K regarded as burn-in. The MCMC was initialised at random values sampled from uniform distributions. Parameter estimation results for all three parameters using adaptive MCMC are shown in Table 2, while Fig. 2 shows histograms of their posterior densities. The ground truth value for each parameter is indicated by a vertical blue line. We can see that only the posterior histograms corresponding to KF2 include the correct estimate for all three parameters in their support. In “Appendix A.15”, we have included traceplots of the MCMC runs for all three parameters, where we can see that the adaptive MCMC leads to a fast convergence for both KF1 and KF2. In order to verify the validity of our results, we have run an extra 100 datasets, each consisting of 40 independent samples and obtained point estimates from KF1 and KF2 using the Nelder–Mead algorithm. The results can be found in “Appendix A.10” and agree with our previous conclusion that inference with KF1 gives inaccurate estimates.

**Table 2:** Mean posterior ± 1 s.d. for $\theta _1, \theta _2, \theta _3$ using an adaptive MCMC

$\theta$	Ground truth	KF1	KF2
$\theta _1$	0.5	$0.480\pm 0.006$	$0.494\pm 0.005$
$\theta _2$	0.0025	$0.0023\pm 5 \times 10^{-5}$	$0.0025\pm 5 \times 10^{-5}$
$\theta _3$	0.3	$0.243\pm 0.010$	$0.298\pm 0.010$

Data were simulated from a Lotka–Volterra model according to the ground truth values

**Fig. 3:**
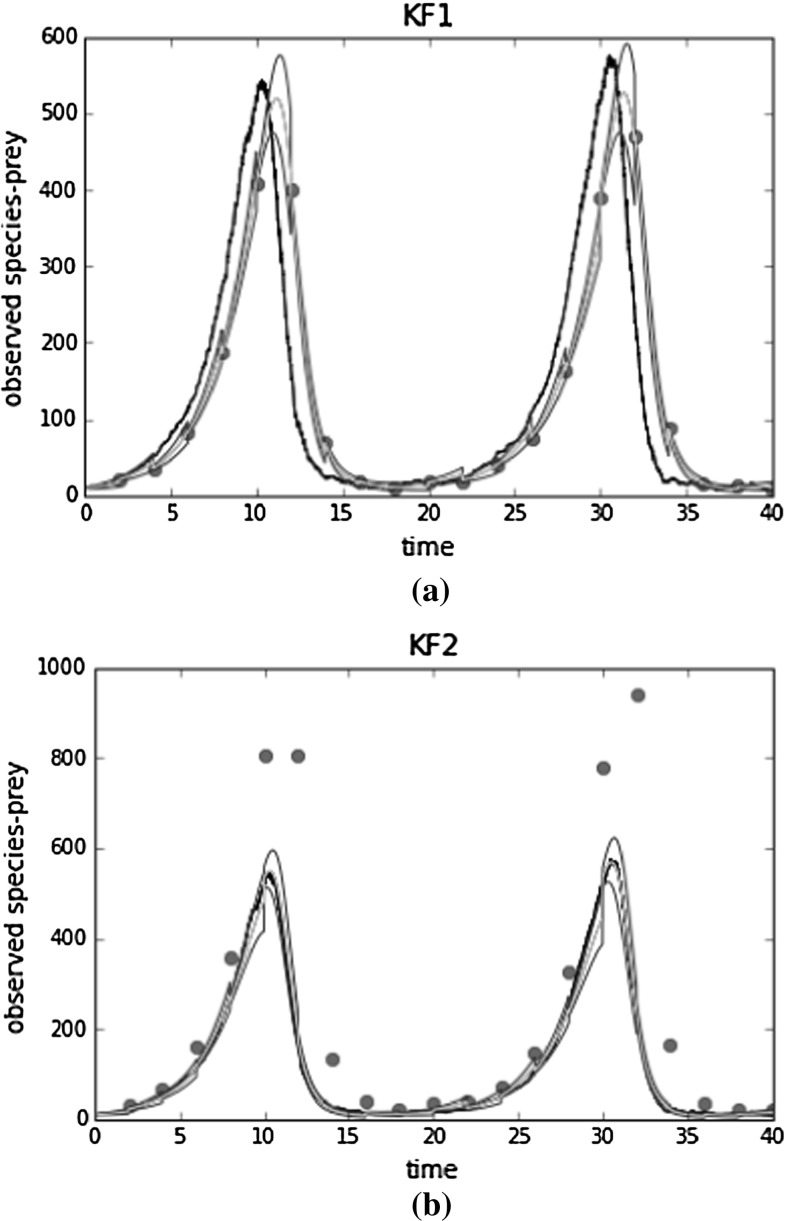
Filtering results for the prey population. Red dots correspond to aggregated observations for KF2 and normalised observations for KF1. The black line represents the actual process. Purple lines represent the mean estimate and green 1 s.d. . **a** Filtering results for the prey population using KF1. **b** Filtering results for the prey population using KF2

Assuming knowledge of the parameter values, we can also use the KF for trajectory inference. In Fig. 3, we demonstrate filtering results for the prey population assuming that we have aggregated data. We simulated a trajectory using $\theta _1 = 0.5 ,\theta _2 = 0.0025,\theta _3 =0.3$ and sampled aggregated data every 2 min. Black lines represent the true trajectory of the populations. We see that the inferred credible region with KF1 does not contain the true underlying trajectory in many places. Note that red dots correspond to normalised (aggregated) observations for KF1 and aggregated observations for KF2, so they do not have the same values. In “Appendix A.16”, we include filtering results for the unobserved predator population.

## Translation inhibition model

In this example, we are interested in inferring the degradation rate of a protein from a translation inhibition experiment. We model the translation inhibition experiment by the following set of reactions where *R* stands for mRNA and *P* for protein:

24a $$\begin{aligned}&R\xrightarrow {{c_P}}R+P \text { (translation)} \end{aligned}$$

24b $$\begin{aligned}&P\xrightarrow {{d_PP/\varOmega }}\oslash \text { (protein degradation)} \end{aligned}$$

The LNA is used, again, as an approximation of the dynamics and the resulting system of ODEs can be found in “Appendix A.11”. Before applying our method to real data from this system, we test the performance on synthetic data simulated using the Gillespie algorithm. We simulated 30 time series (corresponding to 30 different cells), assuming the following values as the ground truth for the kinetic parameters: $c_P = 200$ and $d_P = 0.97$. We further set the initial protein abundance of $m_0$ to 400 molecules. We have scaled the data by a factor $k = 0.03$, so that they are proportional to the original synthetic data and added Gaussian noise with a variance of $s = 0.1$. For this study, we have assumed that data were integrated over 30 min.

Again we use an adaptive MCMC algorithm (Sherlock et al. 2010). Non-informative exponential priors with mean $10^4$ were placed on all parameters. We have adopted the parametarisation used in Komorowski et al. (2009) and Finkenstädt et al. (2013) such as $\widetilde{c}_P = k\cdot c_p$ and $\widetilde{m}_0 = k\cdot m_0$ and worked in the log parameter space. Parameter estimation results for the vector $(c_p,d_p,s,k,m_0)$ using KF1 and KF2 are summarised in Table 3. As we can see, the degradation rates are successfully inferred by both approaches. However, using KF1 leads to an overestimation of $m_0$ and an underestimation of the noise level *s*, which corresponds to a smoother process than the underlying one. MCMC traces from both KF1 and KF2 are presented in Fig. 11.

**Table 3:** Mean posterior ± 1 s.d. for $(c_P, d_P, s, k, m_0)$ using an adaptive MCMC

*c*	GT	KF1	KF2
$c_p$	200	$254.152 \pm 23.3329$	$196.9065 \pm 25.6251$
$d_p$	0.97	$0.9822 \pm 0.0364$	$0.9974 \pm 0.0433$
*s*	0.1	$0.0349 \pm 0.0251$	$0.0995 \pm 0.0093$
*k*	0.03	$0.0236 \pm 0.0017$	$0.0312 \pm 0.0039$
$m_0$	400	$588.9959 \pm 44.0205$	$392.5980 \pm 49.0594$

Data were simulated from a translation inhibition model according to the ground truth (GT) values

**Fig. 4:**
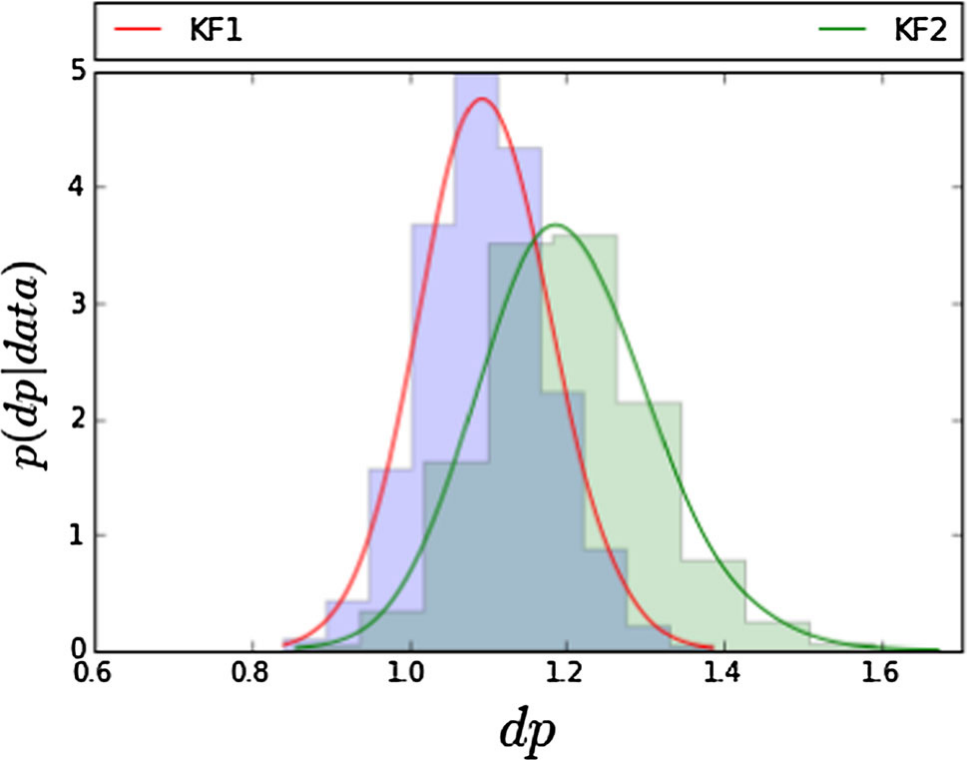
Posterior histograms of degradation rate using KF1 and KF2

**Fig. 6:**
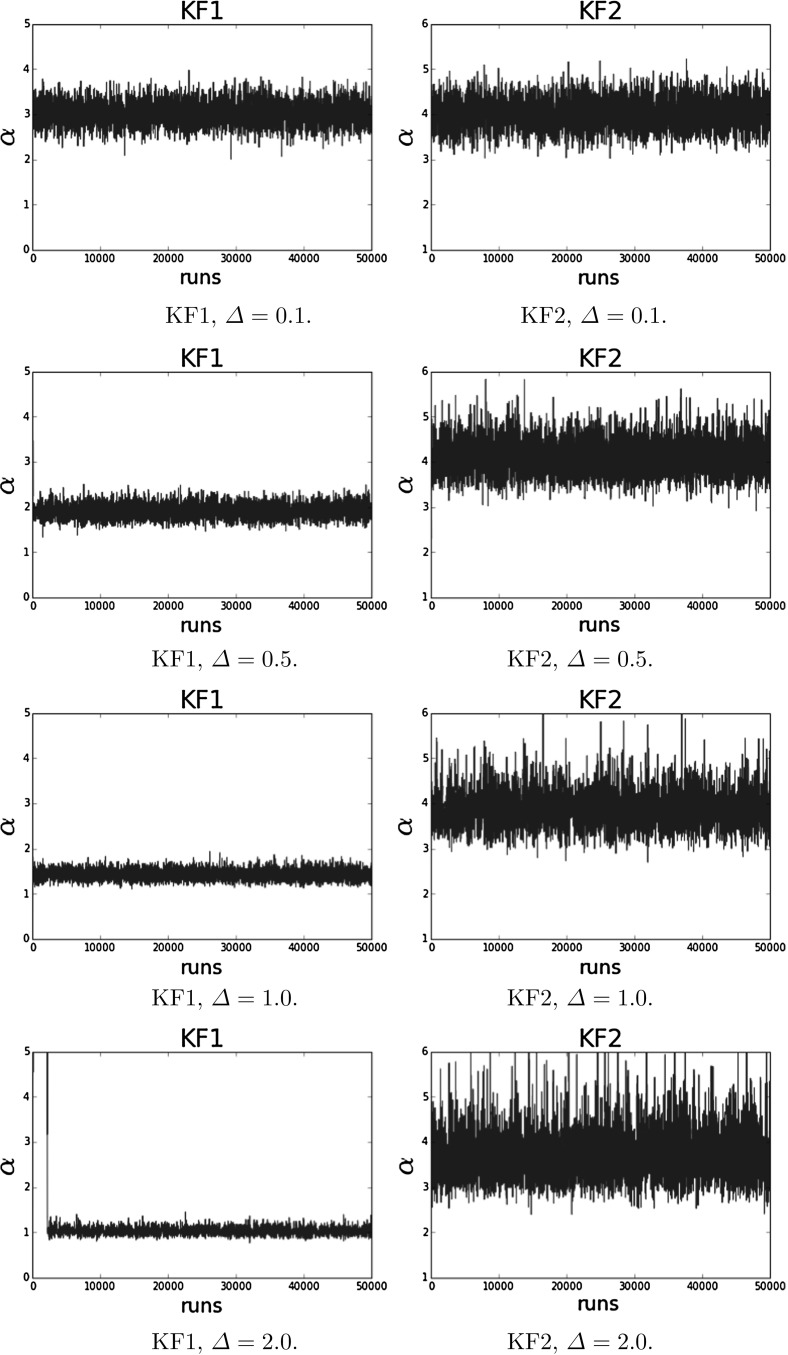
MCMC traces of the posterior of $\alpha$ using a random walk MH for both KF1 and KF2. Ground truth for $\alpha = 4$

**Fig. 7:**
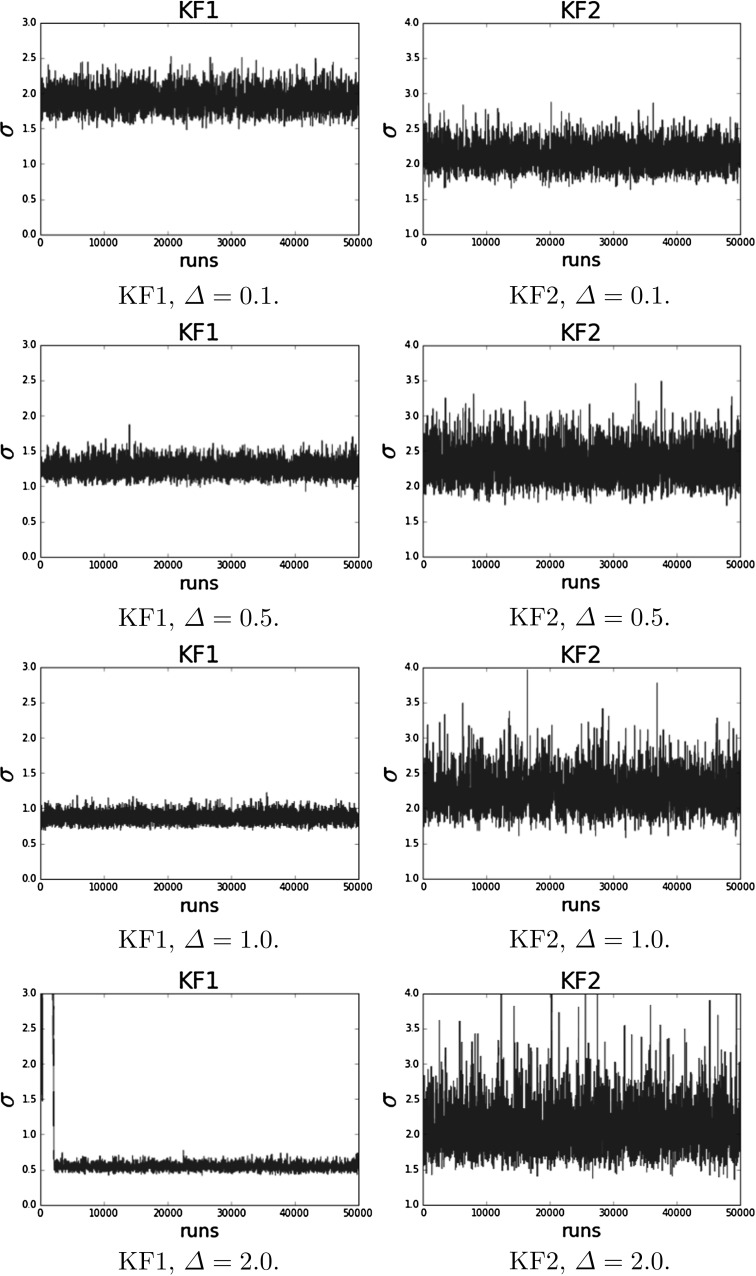
MCMC traces of the posterior of $\sigma$ using a random walk MH for both KF1 and KF2. Ground truth for $\sigma = 2$

**Fig. 11:**
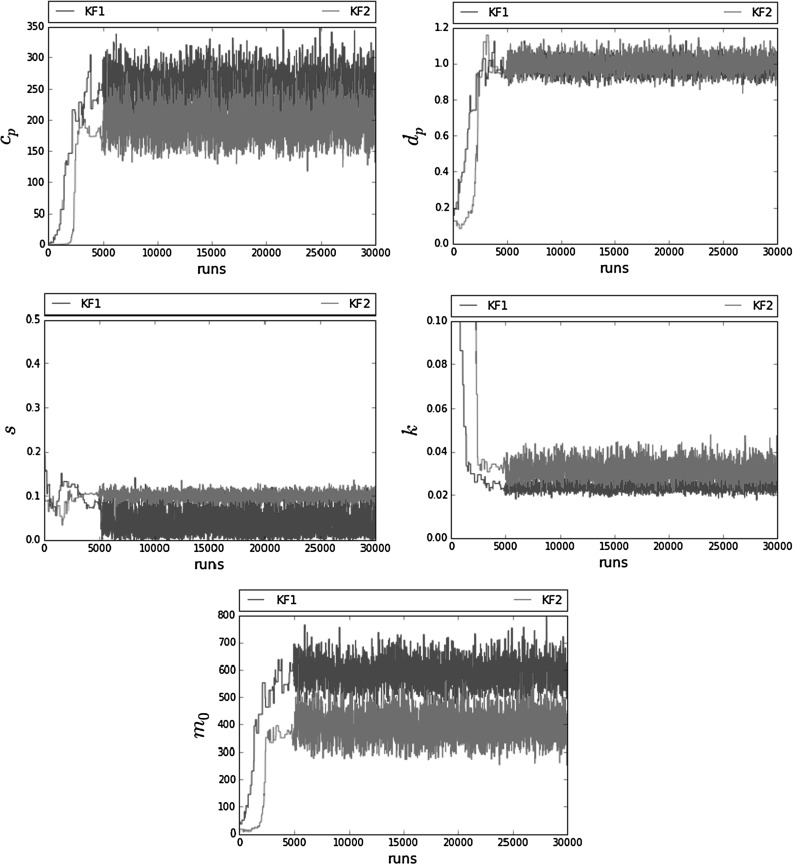
Adaptive MCMC traces of the posterior vector $(c_p,d_p,s,k,m_0)$ using synthetic data with KF1 and KF2

**Fig. 12:**
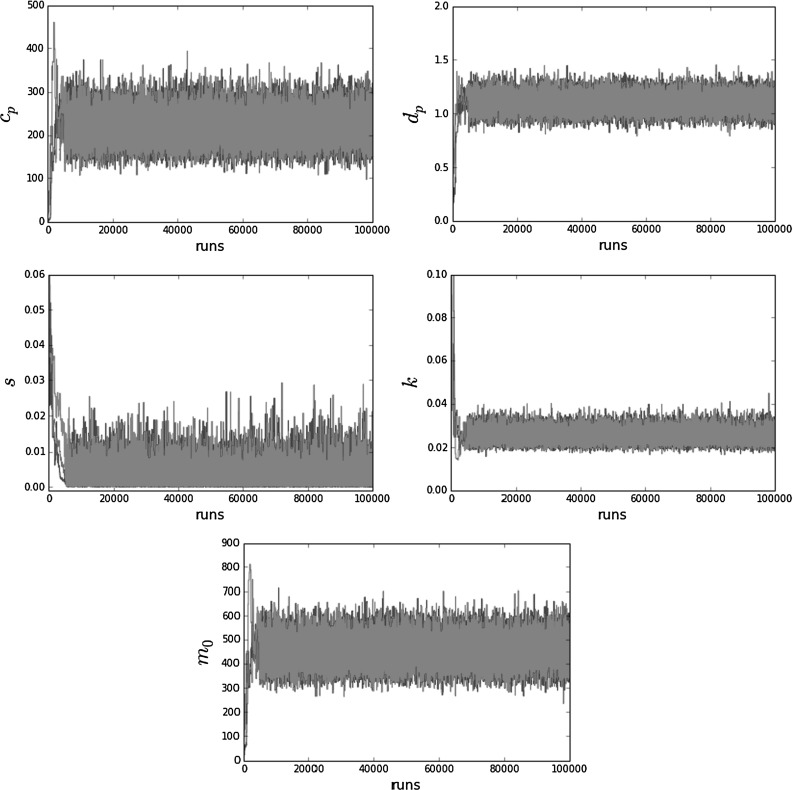
Three MCMC chains of the posterior vector $(c_p,d_p,s,k,m_0)$ using single cell data with KF1

**Fig. 13:**
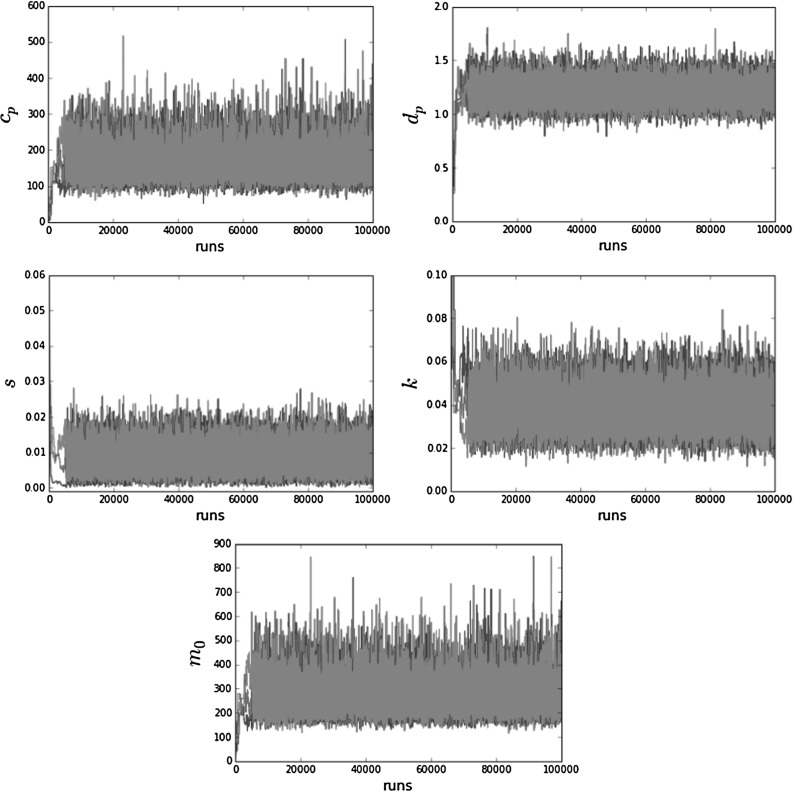
Three MCMC chains of the posterior vector $(c_p,d_p,s,k,m_0)$ using single cell data with KF2

We then applied our model to single cell luciferase data from a subset of 11 pituitary cells (Harper et al. 2011). Parameter estimation results using the same adaptive MCMC are summarised in Table 4. The MCMC was run for 100K iterations out of which 60K were discarded as burn-in. Again, we observe that, using KF1, we get a higher $m_0$ and a slightly lower noise level *s*. Posterior histograms of the degradation rates are shown in Fig. 4. A deterministic approach for fitting the data would give a degradation rate of around 1.02 and, as we can see, this value is included in both histograms of Fig. 4. To check convergence using the Gelman–Rubin statistic, we have run 3 different chains with different initialisations. MCMC traces for both KF1 and KF2 are shown in “Appendix A.18” (Fig. 12 and 13) where we can see that the three chains are very close to each other, corresponding to a Gelman–Rubin statistic close to 1.

**Table 4:** Mean posterior ± 1 s.d. for $(c_P, d_P, s, k, m_0)$ using adaptive MCMC with single cell data obtained from a subset of 11 pituitary cells from a translation inhibition experiment (Harper et al. 2011)

*c*	KF1	KF2
$c_p$	$217.2987 \pm 33.5441$	$169.9254 \pm 43.1153$
$d_p$	$1.1020 \pm 0.0767$	$1.2037 \pm 0.1046$
*s*	$0.0026 \pm 0.0026$	$0.0081 \pm 0.0038$
*k*	$0.0255 \pm 0.0029$	$0.0373 \pm 0.0088$
$m_0$	$449.7679 \pm 53.9760$	$278.2987 \pm 70.6582$

## Discussion

We have presented a Bayesian framework for doing inference using aggregated observations from a stochastic process. Motivated by a systems biology example, we chose to use the LNA to approximate the dynamics of the stochastic system, leading to a linear SDE. We then developed a Kalman filter that can deal with integrated, partial and noisy data. We have compared our new inference procedure to the standard Kalman filter which has previously been applied in systems biology applications approximated using the LNA. Overall, we conclude that the aggregated nature of data should be considered when modelling data, as aggregation will tend to reduce fluctuations and therefore the stochastic contribution of the process may be underestimated.

In Sect. 4, we described the different properties of a stochastic process and its integral in the case of the Ornstein–Uhlenbeck process. We showed that one cannot simply treat the integrated observations as proportional to observations coming from the underlying unintegrated process when carrying out inference. As the aggregation time window increases, parameter estimates using this approach become less accurate and the inferred stationary variance of the process is underestimated. In contrast, our modified KF is able to accurately estimate the model parameters and stationary variance of the process.

In Sect. 5, we have demonstrated the ability of our method to give more accurate results in a Lotka–Volterra model given synthetic aggregated data. In Sect. 6, we looked at a real-world application with data from a translation inhibition experiment carried out in single cells. As the LNA depends on its deterministic part, and in a deterministic system integration is dealt with reasonably well using the simple proportionality constant approach, some of the system parameters, such as the degradation rate, can be inferred reasonably well by the standard non-aggregated data approach. However, neglecting the aggregated nature of the data does lead to a significantly larger estimate of the initial population of molecules even in this simple application. This is consistent with our observation that neglecting aggregation will tend to underestimate the scale of fluctuations as it is the number of molecules that determines the size of fluctuations in this example. In models where noise plays a more critical role, e.g. systems with noise-induced oscillations, the effect of parameter misspecification could have more serious consequences on model-based inferences.

Our proposed inference method can deal with the intrinsic noise inside a cell, measurement noise and temporal aggregation. However, cell populations are highly heterogeneous, and cell-to-cell variability has not been considered in our current inference scheme. It would be possible to deal with cell-to-cell variability using a hierarchical model (Finkenstädt et al. 2013) which could be combined with the integrated data Kalman Filter developed here.

All experiments were carried out on a cluster of 64bit Ubuntu machines with an i5-3470 CPU @ 3.20 GHz x 4 processor and 8 GB RAM. All scripts were run in Spyder (Anaconda 2.5.0, Python 2.7.11, Numpy 1.10.4). Code reproducing the results of the experiments can be found on GitHub https://github.com/maria-myrto/inference-aggregated.

## Figures and Tables

**Fig. 5:**
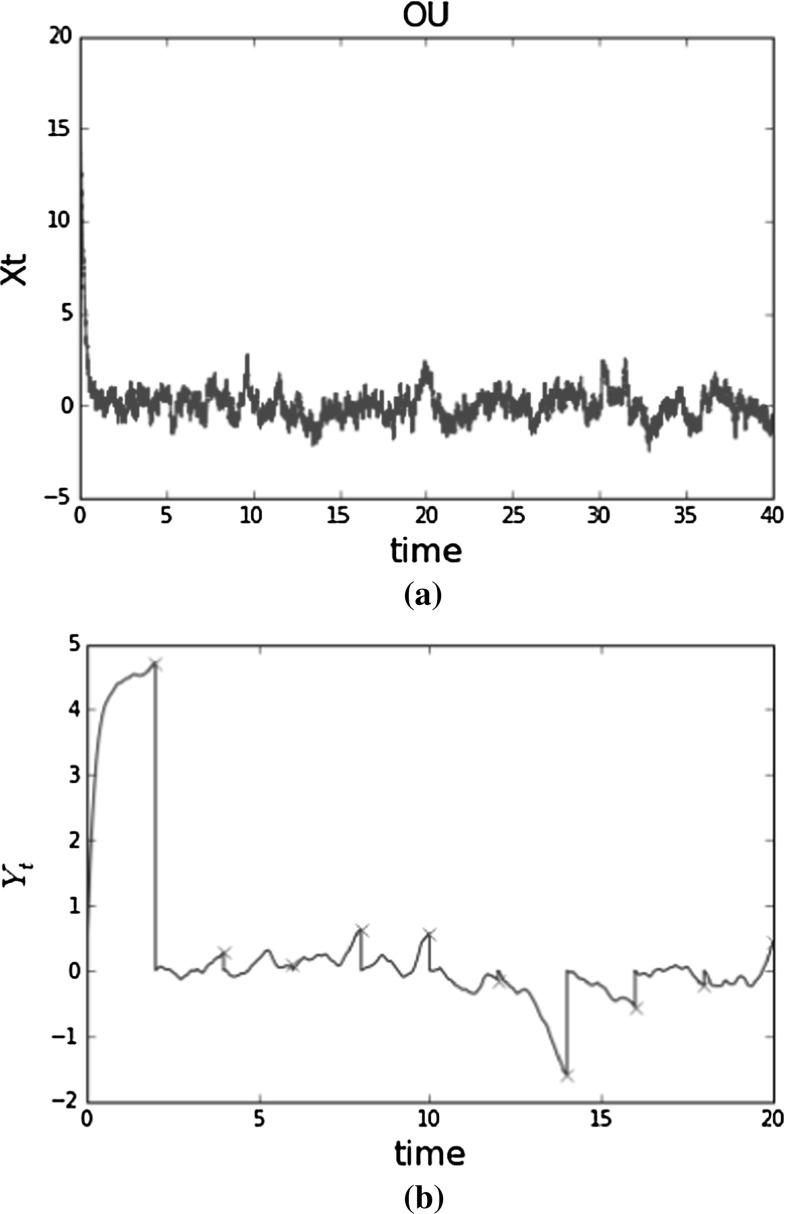
Simulated trajectories from an OU process with $\alpha =4$ and $\sigma =2$, along with the corresponding aggregated process with an integration period of 2 min. For the aggregated process, we assumed observations every 2 min, which are indicated by red crosses. **a** OU process. **b** Aggregated process

**Fig. 8:**
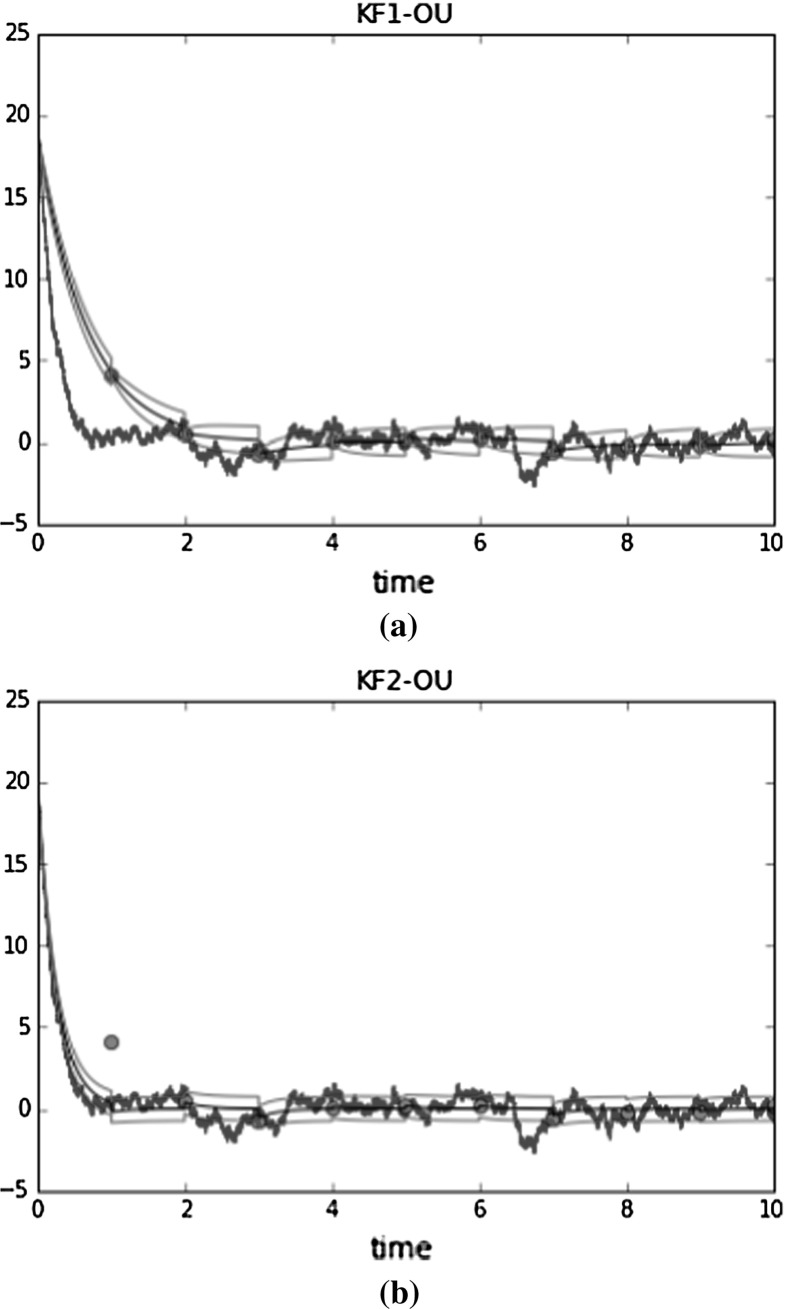
Filtering results from KF1 and KF2 for an OU process with $\alpha = 4.0$ and $\sigma = 2.0$ (blue trace) using aggregated data over an integration period of $\Delta = 1.0$. Black lines correspond to the posterior mean estimate and green lines to 1 s.d.. For inference, we used the estimated parameters from A.7. **a** KF1 ($\Delta =1.0$). **b** KF2 ($\Delta =1.0$). (Color figure online)

**Fig. 9:**
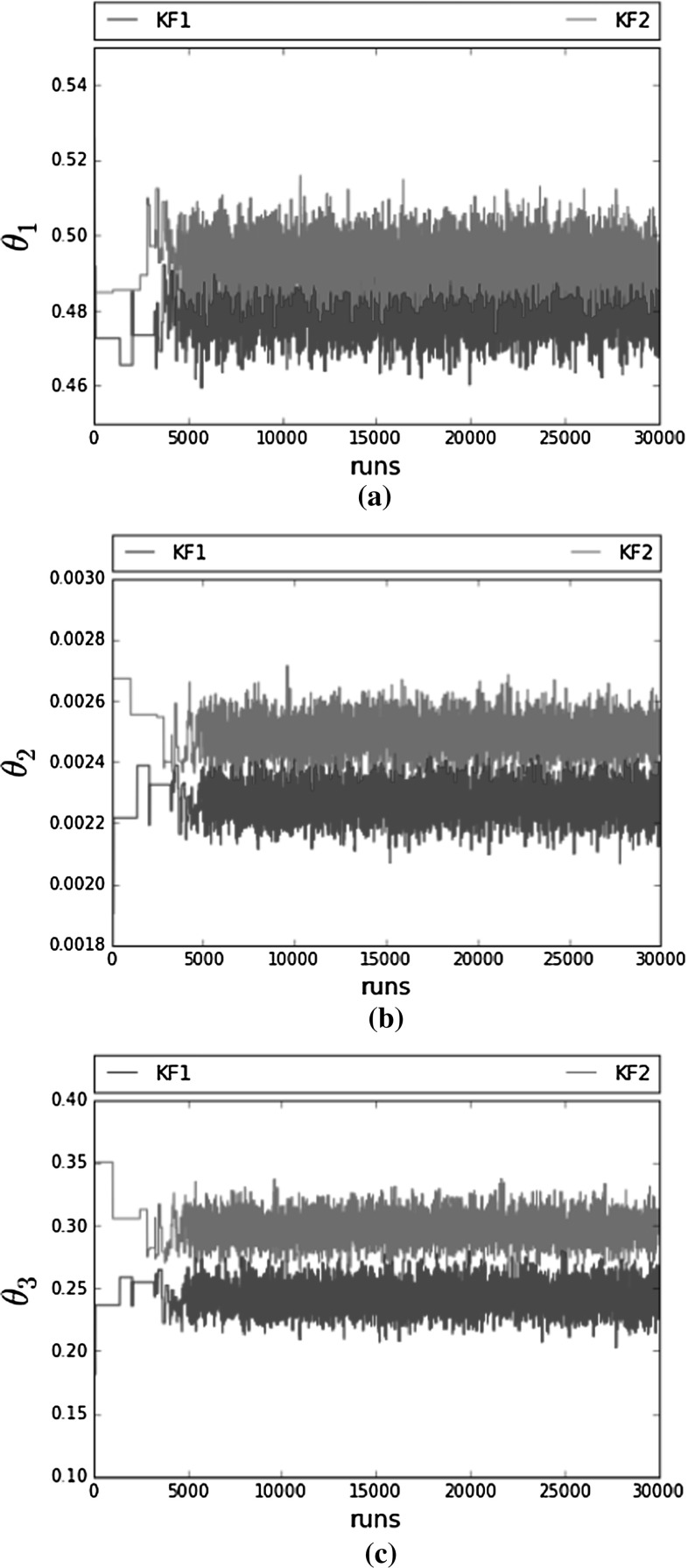
MCMC traceplots for the LV experiment using an adaptive MCMC algorithm. **a** MCMC traceplots for $\theta _1$. **b** MCMC traceplots for $\theta _2$. **c** MCMC traceplots for $\theta _3$

**Fig. 10:**
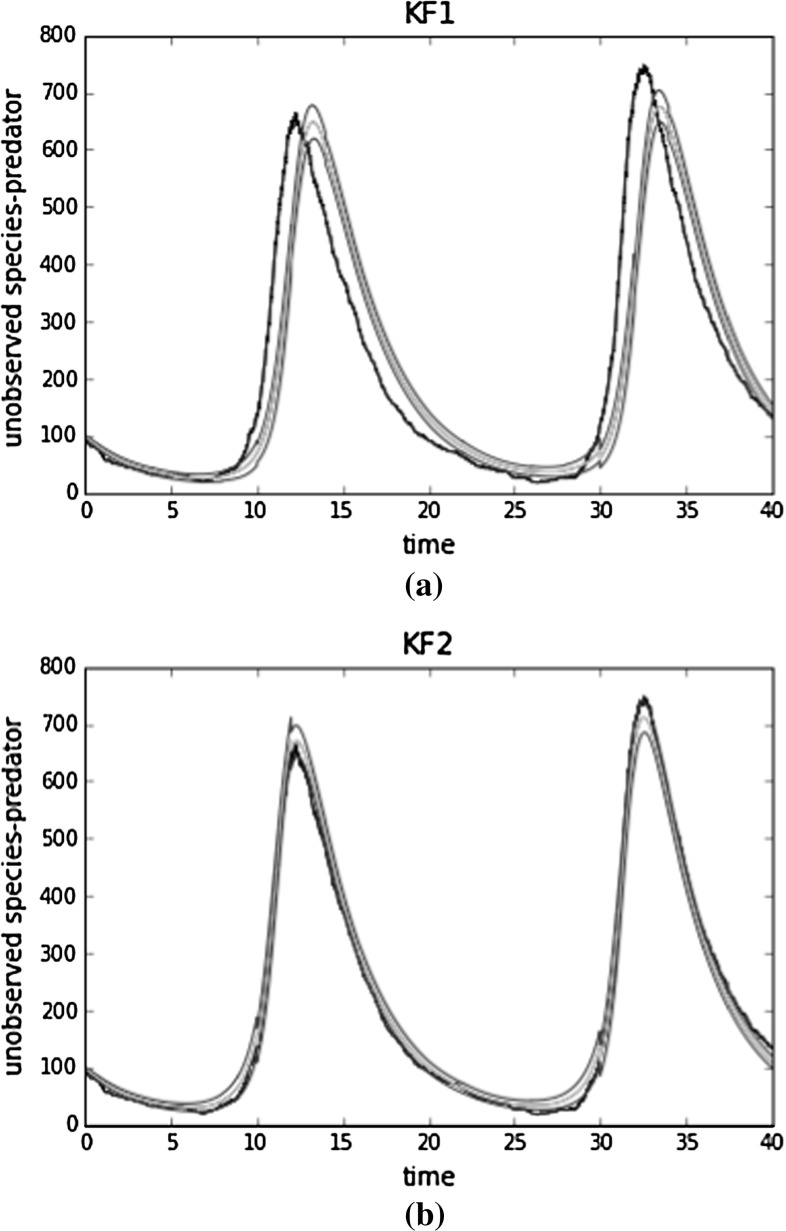
Filtering results for the predator population. Red dots correspond to aggregated observations, and the black line represents the actual process. Purple lines represent the mean estimate and green 1 s.d. . **a** Filtering results for the predator population using KF1. **b** Filtering results for the predator population using KF2

## Appendix

### Mean and variance of the integrated process

We start by computing $\mathbb {E}[Q_t]$, i.e. the **mean** of $Q_t$. We know that:

25 $$\begin{aligned}&d\xi _t = A_t\xi _t\mathrm{d}t + E_t\mathrm{d}W \, , \end{aligned}$$

26 $$\begin{aligned}&dQ_t = \xi _t\mathrm{d}t \Leftrightarrow Q_{t+dt} = Q_t +\xi _t\mathrm{d}t \, . \end{aligned}$$

Averaging Eq. (26), dividing by $\mathrm{d}t$ and letting $\mathrm{d}t\rightarrow 0$, gives us:

27 $$\begin{aligned} \begin{aligned}&\mathbb {E}[Q_{t+\mathrm{d}t}] = \mathbb {E}[Q_t] +\mathbb {E}[\xi _t]\mathrm{d}t\\&\mathbb {E}[Q_{t+\mathrm{d}t}] - \mathbb {E}[Q_t]= \mathbb {E}[\xi _t]\mathrm{d}t\\&\frac{d{E}[Q_{t}]}{\mathrm{d}t} = \mathbb {E}[\xi _t] = 0 \end{aligned} \end{aligned}$$

The mean of $Q_t$ is set to zero, as we have chosen to use the Restarting LNA.

We now need to compute the **covariance** between $Q_t$ and $\xi _t$. Again $\mathbb {E}[Q_t] = 0$ and $\mathbb {E}[\xi _t] = 0$ since we are using the Restarting LNA and thus, the covariance is given by $\mathbb {E}[Q_t\xi _t^T]$. For our derivation, we need to use:

28 $$\begin{aligned} \xi _{t+\mathrm{d}t}^T = \xi _t^T +\xi _t^TA_t^T\mathrm{d}t+E_t^T\mathrm{d}W_t \, . \end{aligned}$$

By multiplying Eqs. (26) and (28) we get:

29 $$\begin{aligned} \begin{aligned} Q_{t+\mathrm{d}t}{\xi _{t+\mathrm{d}t}}^T&=(Q_t+\xi _t\mathrm{d}t)(\xi _t^T+\xi _t^TA^T\mathrm{d}t+E_t^T\mathrm{d}W_t) \\&= Q_t\xi _t^T+Q_t\xi _t^TA_t^T\mathrm{d}t+Q_tE_t^T\mathrm{d}W_t+\\&\quad + \xi _t\xi _t^T\mathrm{d}t+\xi _t\xi _t^TA_t^T\mathrm{d}t\mathrm{d}t+\xi _tE_t^T\mathrm{d}t\mathrm{d}W_t \, . \end{aligned} \end{aligned}$$

Averaging the result (29), retaining terms up to first order in $\mathrm{d}t$, dividing by $\mathrm{d}t$ and letting $\mathrm{d}t\rightarrow 0$, we get:

30 $$\begin{aligned} \begin{aligned}&\mathbb {E}[Q_{t+\mathrm{d}t}{\xi _{t+\mathrm{d}t}}^T]=\mathbb {E}[Q_t\xi _t^T] +\mathbb {E}[Q_t\xi _t^T]A_t^T\mathrm{d}t \\&\qquad \qquad \quad \qquad \qquad +\mathbb {E}[Q_t\mathrm{d}W_t]E_t^T+\mathbb {E}[\xi _t\xi _t^T]\mathrm{d}t \, , \\&\frac{d\mathbb {E}[Q_t\xi _t^T]}{\mathrm{d}t} = \mathbb {E}[Q_t\xi _t^T]A(t)^T + \mathbb {E}[\xi _t\xi _t^T] \, , \\&\frac{d\mathbb {E}[Q_t\xi _t^T]}{\mathrm{d}t} = \mathbb {E}[Q_t\xi _t^T]A(t)^T + V_t \, . \end{aligned} \end{aligned}$$

The **variance** of $Q_t$ is given by $\mathrm {Var}[Q_t]=\mathbb {E}[Q_tQ_t^T]$ since $\mathbb {E}[Q_t] = 0$. We have that,

31 $$\begin{aligned} \begin{aligned} Q_{t+\mathrm{d}t}{Q_{t+\mathrm{d}t}}^T&= (Q_t+\xi _t\mathrm{d}t)(Q_t+\xi _t\mathrm{d}t)^T \, ,\\ Q_{t+\mathrm{d}t}{Q_{t+\mathrm{d}t}}^T&= Q_tQ_t^T+Q_t\xi _t^T\mathrm{d}t+\xi _tQ_t^T\mathrm{d}t+\xi _t\xi _t^T\mathrm{d}t\mathrm{d}t \, . \end{aligned} \end{aligned}$$

By averaging (31), retaining terms up to first order in $\mathrm{d}t$, dividing by $\mathrm{d}t$ and letting $\mathrm{d}t\rightarrow 0$, we get:

32 $$\begin{aligned}&\mathbb {E}[Q_{t+\mathrm{d}t}{Q_{t+\mathrm{d}t}}^T]= \mathbb {E}[Q_tQ_t^T]+\mathbb {E}[Q_t\xi _t^T]\mathrm{d}t+\mathbb {E}[\xi _tQ_t^T]\mathrm{d}t \, , \nonumber \\&\mathbb {E}[Q_{t+\mathrm{d}t}{Q_{t+\mathrm{d}t}}^T] -\mathbb {E}[Q_tQ_t^T]= \mathbb {E}[Q_t\xi _t^T]\mathrm{d}t+\mathbb {E}[\xi _tQ_t^T]\mathrm{d}t \, , \nonumber \\&\frac{\mathrm {Var}[Q_t]}{\mathrm{d}t} = \mathbb {E}[Q_t\xi _t^T] + \mathbb {E}[\xi _tQ_t^T] \, . \end{aligned}$$

### Useful Gaussian identities

Let x and y be jointly Gaussian random vectors:

33 $$\begin{aligned} \begin{bmatrix} x\\y\end{bmatrix} \sim N\left( \begin{bmatrix} \mu _x\\\mu _y\end{bmatrix},\begin{bmatrix}A&C\\C^T&B\end{bmatrix}\right) \end{aligned}$$

Then, the marginal and conditional distributions of *x* (equivalently for *y*) are, respectively (Bishop 2007):

34 $$\begin{aligned}&x \sim N(\mu _x,A) \end{aligned}$$

35 $$\begin{aligned}&x|y \sim N(\mu _x+CB^{-1}(y-\mu _y),A-CB^{-1}C^T) \end{aligned}$$

### Update equations of a discrete Kalman Filter

Using the Gaussian Identities in A.2 we have

36 $$\begin{aligned} \begin{bmatrix} X_i\\y_i\end{bmatrix}|y_{1:(i-1)} \sim N\left( \begin{bmatrix} m_i\\Pm_i\end{bmatrix}, \begin{bmatrix} S_i&S_iP^T\\PS_i&PS_iP^T+R\end{bmatrix}\right) \end{aligned}$$

Since we are working with Gaussians, we know that $X_i|y_{1:i}\sim N(m_i^*,S_i^*)$, and the updated $m_i^*$ and $S_i^*$ are given by:

37 $$\begin{aligned}&m_i^* = m_i + S_iP^T(PS_iP^T+R)^{-1}(y_i - Pm_i) \ , \nonumber \\&S_i^* = S_i + S_iP^T(PS_iP^T+R)^{-1}PS_i \ . \end{aligned}$$

### Analytical solutions for the OU process and its integral

Given an OU process of the following form:

38 $$\begin{aligned} \mathrm{d}X_t = -\alpha X_t\mathrm{d}t + \sigma \mathrm{d}W_t \end{aligned}$$

we can derive its solution according to the general theory for linear SDEs. Since the solution is a Gaussian process, we will only need to define its mean and variance which are given by Eqs. (6, 7). All the ODEs in this case are first-order linear ODEs with constant coefficients, so using for example an integrating factor, we can derive the following solution for an ODE of the form $\frac{\mathrm{d}x}{\mathrm{d}t}+ax=g(t), \quad x(t=0)=x_0$:

39 $$\begin{aligned} x_t = e^{-a(t-t_0)}x_0+\int _{t_0}^t e^{-a(t-\tau )}g(\tau )d\tau . \end{aligned}$$

For the mean we get from Eq. (6):

40 $$\begin{aligned} \begin{aligned}&\frac{\mathrm{d}m_t}{\mathrm{d}t} = -\alpha m_t, \quad m_{t_0}=m_0\Rightarrow \\&m_t = m_0e^{-a(t-t_0)} \end{aligned} \end{aligned}$$

For the variance we have the following:

41 $$\begin{aligned} \begin{aligned}&\frac{\mathrm{d}V_t}{\mathrm{d}t} = -2\alpha V_t + \sigma ^2, \quad V_{t_0}=V_0 \Rightarrow \\&V_t = e^{-2\alpha (t-t_0)}V_0+\int _{t_0}^te^{-2\alpha (t-\tau )} \sigma ^2d\tau \Rightarrow \\&V_t = e^{-2\alpha (t-t_0)}V_0+\frac{\sigma ^2}{2\alpha }(1-e^{-2\alpha (t-t_0)}) \end{aligned} \end{aligned}$$

**Table 5:** Average of mean posterior ± 1 s.d. over 10 different datasets for $\alpha$ and $\sigma$ using a Metropolis–Hastings algorithm

$\Delta$	KF	$\alpha$	$\sigma$
0.1	KF1	$3.081\pm 0.258$	$1.670\pm 0.209$
0.5	KF1	$1.956\pm 0.153$	$1.199\pm 0.125$
1.0	KF1	$1.493\pm 0.112$	$0.799\pm 0.088$
2.0	KF1	$1.064\pm 0.090$	$0.485\pm 0.046$
0.1	KF2	$4.171\pm 0.417$	$1.974\pm 0.208$
0.5	KF2	$4.121\pm 0.377$	$2.068\pm 0.257$
1.0	KF2	$4.123\pm 0.445$	$2.083\pm 0.283$
2.0	KF2	$4.208\pm 0.783$	$2.091\pm 0.371$

Data were simulated from an OU process with $\alpha = 4$ and $\sigma = 2$

**Table 6:** Nelder–Mead results for $\theta _1,\theta _2,\theta _3$. The median values across 100 datasets are shown in the third and fourth column for KF1 and KF2, respectively

$\theta$	Ground truth	KF1 Median[LQ,UQ]	KF2 Median[LQ,UQ]
$\theta _1$	0.5	0.48160 [0.47770,0.48651]	0.49746 [0.49278,0.50122]
$\theta _2$	0.0025	0.00227 [0.00222,0.00232]	0.00248[0.00244,0.00254]
$\theta _3$	0.3	0.24773 [0.23927,0.25797]	0.30047[0.29320,0.31061]

Lower and upper quartiles are shown in brackets

For the solution of the integrated OU process $\mathrm{d}Y_t/\mathrm{d}t = X_t$, we need to calculate its mean, covariance and variance given by Eqs. (12), (13) and (14). The initial conditions for these ODEs will be set to 0, since at each observation point the integrated process starts from 0. For clarity, we will use the results *A*, *B*, *C* from “Appendix A.5”.

First we find the mean:

42 $$\begin{aligned} \begin{aligned}&\frac{d\mathbb {E}_t}{\mathrm{d}t}= m_t = m_0e^{-\alpha (t-t_0)}, \quad \mathbb {E}(t_0) = 0 \Rightarrow \\&\mathbb {E}_t = \int _{t_0}^tm_0e^{-\alpha (\tau -t_0)}d\tau {\mathop {\Rightarrow }\limits ^{A}}\\&\mathbb {E}_t = \frac{m_0}{\alpha }(1-e^{-\alpha (t-t_0)}) \end{aligned} \end{aligned}$$

For the covariance, we first calculate from Eq. (13):

43 $$\begin{aligned} \begin{aligned}&\frac{d\mathbb {E}[X_tY_t]}{\mathrm{d}t} = -\alpha \mathbb {E}[X_tY_t] +\mathbb {E}[{X_t}^2], \quad \mathbb {E}[X_0Y_0] = 0 {\Rightarrow }\\&\mathbb {E}[X_tY_t] = \int _{t_0}^t\mathbb {E}[{X_t}^2]e^ {-\alpha (t-\tau )}d\tau {\mathop {\Rightarrow }\limits ^{\mathbb {E}[{X_t}^2] =V_t+{m_t}^2}}\\&\mathbb {E}[X_tY_t] = \int _{t_0}^t\left( \left( m_0^2-\frac{\sigma ^2}{2\alpha }+V_0 \right) e^{-2\alpha (\tau -t_0)}\right. \\&\left. \qquad \qquad \quad +\frac{\sigma ^2}{2\alpha }\right) e^{-\alpha (t-\tau )}d\tau {\mathop {\Rightarrow }\limits ^{A,C}}\\&\mathbb {E}[X_tY_t] = \frac{\sigma ^2}{2\alpha ^2} (1-e^{-\alpha (t-t_0)})+\\&\frac{1}{\alpha }\left( m_0^2-\frac{\sigma ^2}{2\alpha }+V_0\right) (e^{-\alpha (t-t_0)}-e^{-2\alpha (t-t_0)}) \end{aligned} \end{aligned}$$

Now the covariance can be calculated from:

44 $$\begin{aligned} \begin{aligned}&\mathrm{Cov}(X_t,Y_t) = \mathbb {E}[X_tY_t]-m_t\mathbb {E}_t{\Rightarrow }\\&\mathrm{Cov}(X_t,Y_t) = \\&=\frac{\sigma ^2}{2\alpha ^2}+\left( -\frac{\sigma ^2}{\alpha ^2} +\frac{V_0}{\alpha }\right) e^{-\alpha (t-t_0)}+\left( \frac{\sigma ^2}{2\alpha ^2} -\frac{V_0}{\alpha }\right) e^{-2\alpha (t-t_0)} \end{aligned} \end{aligned}$$

For the variance, we need to calculate:

45 $$\begin{aligned} \begin{aligned}&\frac{d\mathbb {E}[{Y_t}^2]}{\mathrm{d}t} = 2\mathbb {E}[X_tY_t],\quad \mathbb {E}[{Y_0}^2]=0{\Rightarrow }\\&\mathbb {E}[{Y_t}^2] = 2\int _{t_0}^t\mathbb {E}[X_\tau Y_\tau ] d\tau {\mathop {\Rightarrow }\limits ^{(43),B}}\\&\mathbb {E}[{Y_t}^2] = \frac{{m_0}^2}{\alpha ^2}(1-2e^{-\alpha (t-t_0)}+e^{-2\alpha (t-t_0)}) \end{aligned} \end{aligned}$$

Now we can derive the variance:

46 $$\begin{aligned} \begin{aligned} \mathrm {Var}[y_t]&= \mathbb {E}[{Y_t}^2]- {\mathbb {E}_t}^2{\Rightarrow }\\ \mathrm {Var}[y_t]&= \frac{\sigma ^2}{\alpha ^2}(t-t_0) +(\frac{\sigma ^2}{2\alpha ^3}-\frac{V_0}{\alpha ^2})(1-e^{-2\alpha (t-t_0)})\\&\quad +2(-\frac{\sigma ^2}{\alpha ^3}+\frac{V_0}{\alpha ^2})(1-e^{-\alpha (t-t_0)}) \end{aligned} \end{aligned}$$

### Frequently used integrals for part (A.4)

47 $$\begin{aligned} A= & {} \int _{t_0}^te^{-\alpha (\tau -t_0)}d\tau = \frac{1}{\alpha }(1-e^{-\alpha (t-t_0)}) \end{aligned}$$

48 $$\begin{aligned} B= & {} \int _{t_0}^te^{-2\alpha (\tau -t_0)}d\tau = \frac{1}{2\alpha }(1-e^{-2\alpha (t-t_0)}) \end{aligned}$$

49 $$\begin{aligned} C= & {} \int _{t_0}^te^{-\alpha (t-\tau )}e^{-2\alpha (\tau -t_0)} d\tau \nonumber \\= & {} \frac{1}{\alpha }(e^{-\alpha (t-t_0)}-e^{-2\alpha (t-t_0)}) \end{aligned}$$

### Exact updating formula of OU process

The OU process $\mathrm{d}X_t = -\alpha X_t\mathrm{d}t + \sigma \mathrm{d}W_t$ admits an exact update formula given by Gillespie (1992):

50 $$\begin{aligned} X_{t+\mathrm{d}t} = X_te^{-\alpha \mathrm{d}t} + \sqrt{\sigma ^2\frac{1}{2 \alpha }e^{-2\alpha dt}} N(0,1), \end{aligned}$$

### Average over 10 datasets—OU example

See Table 5.

### LNA for Lotka–Volterra model

The Lotka–Volterra model (23) gives rise to the stoichiometry matrix,

51 $$\begin{aligned} S = \begin{bmatrix} 1&-1&0\\ 0&1&-1 \end{bmatrix} , \end{aligned}$$

with transition rates,

52 $$\begin{aligned} h(X) = \begin{bmatrix} \theta _1X_1\\ \theta _2X_1X_2\\\theta _3X_2 \end{bmatrix}. \end{aligned}$$

The following matrices need to be computed:

53 $$\begin{aligned}&F = \begin{bmatrix} \theta _1&0\\ \theta _2y_2&\theta _2y_1\\ 0&\theta _3 \end{bmatrix} , \end{aligned}$$

54 $$\begin{aligned}&SF^T= A= \begin{bmatrix} \theta _1 -\theta _2y_2&-\theta _2y_1\\ \theta _2y_2&\theta _2y_1 -\theta _3 \end{bmatrix} , \end{aligned}$$

55 $$\begin{aligned}&Sdiag(h(y_t))S^T = EE^T\nonumber \\&=\begin{bmatrix} \theta _1y_1 +\theta _2y_1y_2&-\theta _2y_1y_2\\ -\theta _2y_1y_2&\theta _2y_1y_2 +\theta _3y_2 \end{bmatrix} , \end{aligned}$$

The macroscopic rate equations are now given by:

56 $$\begin{aligned} \frac{\mathrm{d}y_1}{\mathrm{d}t}= & {} \theta _1y_1 - \theta _2y_1y_2 \end{aligned}$$

57 $$\begin{aligned} \frac{\mathrm{d}y_2}{\mathrm{d}t}= & {} \theta _2y_1y_2 - \theta _3y_2 \end{aligned}$$

For the diffusion terms, we only need to compute the variance of the resulting Gaussian process since we restart the stochastic part at each observation point in accordance with (Fearnhead et al. 2014).

58 $$\begin{aligned} \begin{aligned} \frac{\mathrm{d}V}{\mathrm{d}t}&= VA^T + EE^T +AV = \\&\quad \begin{bmatrix} V_{11}&V_{12}\\ V_{21}&V_{22} \end{bmatrix} \begin{bmatrix} \theta _1 -\theta _2y_2&\theta _2y_2\\ -\theta _2y_1&\theta _2y_1 -\theta _3 \end{bmatrix} + \\&\quad + \begin{bmatrix} \theta _1y_1 +\theta _2y_1y_2&-\theta _2y_1y_2\\ -\theta _2y_1y_2&\theta _2y_1y_2 +\theta _3y_2 \end{bmatrix}+\\&\quad + \begin{bmatrix} \theta _1 -\theta _2y_2&-\theta _2y_1\\ \theta _2y_2&\theta _2y_1 -\theta _3 \end{bmatrix} \begin{bmatrix} V_{11}&V_{12}\\ V_{21}&V_{22} \end{bmatrix} \end{aligned} \end{aligned}$$

*V* is a symmetric matrix so $V_{12} = V_{21}$. So:

59 $$\begin{aligned} \frac{\mathrm{d}V_{11}}{\mathrm{d}t}= & {} 2V_{11}(\theta _1-\theta _2y_2) - 2V_{12}\theta _2y_1 + \theta _2y_1y_2 +\theta _1y_1\nonumber \\ \end{aligned}$$

60 $$\begin{aligned} \frac{\mathrm{d}V_{12}}{\mathrm{d}t}= & {} V_{12}(\theta _2y_1-\theta _3+\theta _1 -\theta _2y_2) +V_{11}\theta _2y_2 -\theta _2y_1V_{22} \nonumber \\&- \theta _2y_1y_2 \end{aligned}$$

61 $$\begin{aligned} \frac{\mathrm{d}V_{22}}{\mathrm{d}t}= & {} 2V_{22}(\theta _2y_1 -\theta _3) + 2V_{12}\theta _2y_2 + \theta _2y_1y_2 +\theta _3y_2 \end{aligned}$$

The integrated process $\mathrm{d}Y_t = X_t\mathrm{d}t$ follows Eqs. (10),(13),(14). The deterministic part is given by:

62 $$\begin{aligned} \frac{\mathrm{d}I_1}{\mathrm{d}t} = y_1 \ , \quad \frac{\mathrm{d}I_2}{\mathrm{d}t} = y_2 \ . \end{aligned}$$

The ODEs for its integrated variance and covariance with the underline process $X_t$ are given below, where $$\mathrm {Cov}(YX^T) = C_t= \begin{bmatrix} C_{11}&C_{12}\\ C_{21}&C_{22} \end{bmatrix} $$ and $\mathrm {Var}(Y) = G_t$:

63 $$\begin{aligned} \frac{\mathrm{d}C}{\mathrm{d}t} =\begin{bmatrix} C_{11}&C_{12}\\ C_{21}&C_{22} \end{bmatrix} \begin{bmatrix} \theta _1 -\theta _2y_2&\theta _2y_2\\ -\theta _2y_1&\theta _2y_1 -\theta _3 \end{bmatrix} + \begin{bmatrix} V_{11}&V_{12}\\ V_{12}&V_{22} \end{bmatrix} \, , \end{aligned}$$

such as,

64 $$\begin{aligned}&\frac{\mathrm{d}C_{11}}{\mathrm{d}t} = (\theta _1-\theta _2y_2)C_{11} - \theta _2y_1C_{12} + V_{11} \end{aligned}$$

65 $$\begin{aligned}&\frac{\mathrm{d}C_{12}}{\mathrm{d}t} = \theta _2y_2C_{11}+(\theta _2y_1-\theta _3)C_{12} + V_{12} \end{aligned}$$

66 $$\begin{aligned}&\frac{\mathrm{d}C_{21}}{\mathrm{d}t} =(\theta _1-\theta _2y_1)C_{21} - \theta _2y_1C_{22} + V_{12} \end{aligned}$$

67 $$\begin{aligned}&\frac{\mathrm{d}C_{22}}{dt} = \theta _2y_2C_{21}+(\theta _2y_1-\theta _3)C_{22} + V_{22}\end{aligned}$$

68 $$\begin{aligned}&\frac{\mathrm{d}G}{\mathrm{d}t} = C_t + {C_t}^T \end{aligned}$$

### Adaptive MCMC

According to the specific adaptive MH (Sherlock et al. 2010), the new state $\theta ^*$ is sampled from a mixture of Gaussians:

69 $$\begin{aligned} \theta ^* = {\left\{ \begin{array}{ll} N(\theta _t,\varSigma _0), &{} \text{ w.p. } \, \delta \\ N(\theta _t,\lambda \varSigma _t), &{} \text{ w.p. } \, 1-\delta \end{array}\right. } \end{aligned}$$

$\varSigma _t$ corresponds to the sampled variance up to iteration *t* and is estimated after enough samples have been accepted. The parameter $\delta \in (0,1)$ and is defined by the user, we have chosen a value of 0.05. The scaling factor $\lambda$ can either be fixed (Roberts and Rosenthal 2009) or be tuned (Sherlock et al. 2010). This algorithm targets an acceptance rate of $\approx 0.3$.

### Nelder Mead results for LV model

See Table 6.

### LNA for translation inhibition model

The following model is being assumed where R and P stand for the (numbers of) gene mRNA and protein, respectively:

70a $$\begin{aligned}&R\xrightarrow {{c_P}}R+P \text { (translation)}\end{aligned}$$

70b $$\begin{aligned}&P\xrightarrow {{d_PP/\varOmega }}\oslash \text { (protein degradation)} \end{aligned}$$

The above equations result in the following stoichiometry matrix:

71 $$\begin{aligned} S = \begin{bmatrix} 1&-1 \end{bmatrix} , \end{aligned}$$

and the transition rates are :

72 $$\begin{aligned} h(x,t) = \begin{bmatrix} c_P\\ d_Pp \end{bmatrix} , \end{aligned}$$

The required matrices are calculated below:

73 $$\begin{aligned}&F = \begin{bmatrix} 0&d_P \end{bmatrix} , \end{aligned}$$

74 $$\begin{aligned}&SF^T= A= \begin{bmatrix} -d_P \end{bmatrix} ,\end{aligned}$$

75 $$\begin{aligned}&\mathrm{Sdiag}(h(y_t,\theta ))S^T = EE^T = \begin{bmatrix} c_P + d_Pp\end{bmatrix}, \end{aligned}$$

The deterministic part is now given by:

76 $$\begin{aligned} \frac{\mathrm{d}p}{\mathrm{d}t} = c_P - d_Pp \end{aligned}$$

The stochastic part is given by the (restarting) LNA where we have dropped the dependency of $M_t,V_t$ from time:

77 $$\begin{aligned} \mathrm{d}M_p = -d_pM_p\mathrm{d}t + \sqrt{c_P + d_Pp}\mathrm{d}W_t \end{aligned}$$

resulting in the following ODE for the stochastic variance:

78 $$\begin{aligned} \frac{\mathrm{d}V_p}{\mathrm{d}t} = -2d_pV_p + c_P + d_Pp \end{aligned}$$

For the integrated process, we get the following according to Eqs. (10),(13) and (14) (Fig. 5). First the deterministic part is given by:

79 $$\begin{aligned} \frac{\mathrm{d}I_p}{\mathrm{d}t} = p, \end{aligned}$$

The stochastic part is given by:

80 $$\begin{aligned} \frac{\mathrm{d}Q_p}{\mathrm{d}t} = M_p, \end{aligned}$$

resulting in the following ODEs for its integrated variance and covariance with the unintegrated process:

81 $$\begin{aligned} \frac{dCov(Q_pM_p^T)}{\mathrm{d}t}= & {} -d_pCov(Q_pM_p^T) + V_p \end{aligned}$$

82 $$\begin{aligned} \frac{d\mathrm {Var}(Q_p)}{\mathrm{d}t}= & {} 2Cov(Q_pM_p^T) \end{aligned}$$

### OU and aggregated OU process

See Fig. 5.

### OU traceplots

Figures 6 and 7 show samples of the OU parameters during the MCMC runs.

### Filtering results for OU process using aggregated data

See Fig. 8.

### MCMC traces from LV experiment

See Fig. 9.

### Filtering results for the predator population

See Fig. 10.

### MCMC traces for Translation inhibition example with synthetic data

See Fig. 11.

### MCMC traces for translation inhibition example with single cell data

See Figs. 12 and 13
